# Hyperactivation of RAB5 disrupts the endosomal Rab cascade leading to endolysosomal dysregulation in Down syndrome: A necessary role for increased *APP* gene dose

**DOI:** 10.1002/alz.70046

**Published:** 2025-05-07

**Authors:** Xu‐Qiao Chen, Xinxin Zuo, Ann Becker, William C. Mobley

**Affiliations:** ^1^ Department of Neurosciences University of California San Diego La Jolla California USA

**Keywords:** Alzheimer's disease, APP, cathepsin, down syndrome, Dp16 mouse, endosomal rab cascade, GEF, RAB5

## Abstract

**INTRODUCTION:**

Down syndrome (DS) markedly increases the risk of Alzheimer's disease (DS‐AD), but the role of RAB5 hyperactivation in its pathogenesis remains unclear.

**METHODS:**

Postmortem brain samples from individuals with DS, with and without AD, and a partial trisomy 21 case with only two amyloid precursor protein (*APP*) gene copies, were examined for endosomal Rabs, their guanine‐nucleotide exchange factor (GEF) and GTPase activating protein (GAP) levels, and lysosomal cathepsins. Analysis extended to the Dp16 DS mouse model. The role of RAB5 hyperactivation in disrupting the endolysosomal system was explored using primary neurons.

**RESULTS:**

We observed widespread endolysosomal dysregulation in DS and Dp16 brains, requiring increased *APP* gene dose. RAB5 hyperactivation resulted in increased activation of endosomal Rabs, including RABs 7 and 11, and increased recruitment of Rabs and their GEFs to early endosomes as well as the levels of lysosomal cathepsins.

**DISCUSSION:**

These findings suggest that *APP* dose‐driven RAB5 hyperactivation disrupts endosomal Rab cascades and endosome maturation in DS.

**Highlights:**

- There is widespread disruption of the endolysosomal network in the Down syndrome (DS) brain and in the Dp16 mouse model brain.
- Amyloid precursor protein (*APP*) gene dose was necessary for increases in endosomal Rab activity and lysosomal cathepsins in both human and mouse brains.
- Changes in endosomal Rabs 7 and 11 were linked to increases in their guanine‐nucleotide exchange factors (GEFs) and GEF/GTPase activating protein (GAP) ratios.
- Mechanistic studies demonstrated essential roles for the beta‐C‐terminal fragment (β‐CTF) of APP acting through hyperactivation of RAB5 to increase early endosomal membrane binding of the GEFs for downstream endosomal Rabs.
- RAB5 acts as the central hub for disruptions in endolysosomal function in DS.

## 1 BACKGROUND

Alzheimer's disease (AD), the most commonly diagnosed form of dementia, typically presents with late‐onset short‐term memory loss and other cognitive deficits, and it can be accompanied by mood changes and impaired daily activities.^1^ Although the pathogenesis of AD is complex, disease‐modifying treatments targeting amyloid beta (Aβ) deposits have been approved.^2^ This has further motivated research to elucidate underlying mechanisms and identify new treatment targets.

Down syndrome (DS), a genetic anomaly involving trisomy of human chromosome 21, represents an important context for AD research due to the high incidence of early‐onset AD in DS (DS‐AD) adults.^3^, ^4^ We estimate that about 70,000 adults with DS in the United States are at risk for DS‐AD, which shares with AD clinical features and neuropathological hallmarks, including Aβ plaques and neurofibrillary tangles,^5^ but with differences in age‐dependent changes in the levels of Aβ and tau prion‐like species.^6^

A less‐explored hallmark of AD is the enlarged early endosomes (EEs) and increased RAB5 activity. RAB5, a small GTPase Rab protein family member, regulates endosomal trafficking and intracellular signaling.^7^ The endolysosomal network (ELN) includes subcellular compartments marked by specific Rab proteins. Although RAB5 is a specific marker of EEs and mediates early events in the endocytic pathway, RAB7, found on late endosomes (LEs), autophagosomes, and lysosomes, facilitates the maturation of LEs and autophagosomes and their fusion with lysosomes. RAB11 is involved in slow endocytic recycling through recycling endosomes (REs); and RAB4 mediates rapid endocytic recycling from EEs.^8^ Rab activity is regulated by guanosine 5′‐triphosphate (GTP)/guanosine 5'‐diphosphate (GDP) cycles. Guanine‐nucleotide exchange factors (GEFs) catalyze the exchange of GTP for GDP while GTPase‐activating proteins (GAPs) mediate the hydrolysis of GTP to GDP.^8^, ^9^

RAB5‐mediated dysregulation of the ELN could compromise cellular structure and function and contribute to AD and DS‐AD pathogenesis.^10^, ^11^ Results that focused on the pathogenesis of sporadic AD revealed that elevated levels of RAB5 protein led to its hyperactivation and EE enlargement.^12^, ^13^, ^14^ Another mechanism pointed to increased levels of the amyloid precursor protein (APP) beta‐C‐terminal fragment (β‐CTF) interacting with the APPL1 (the adaptor protein containing a pleckstrinhomology [PH] domain, phosphotyrosine binding [PTB] domain, and leucine zipper motif 1) scaffolding protein to increase RAB5 binding and activity on EEs in sporadic AD, familial AD (FAD), or DS‐AD.^15^, ^16^, ^17^, ^18^, ^19^ In addition to APP, other proteins have also been implicated in the enlargement of EE in AD and DS, including SorLA^20^ and synaptojanin1.^21^ High‐resolution light imaging and ultrastructural methods have recently reported that rather than being increased in size, the clustering of EEs explains the apparent increase in size in the setting of DS.^22^ Whether enlarged or clustered, changes in EE morphology and significantly increased levels of GTP‐RAB5 point to dysregulation of RAB5 in AD and DS.^16^, ^17^, ^23^ The importance of RAB5 hyperactivation was underscored by findings indicating that forced overexpression of RAB5 induced ELN dysregulation, neuronal dysfunction, and degenerative phenotypes.^24^

To explore the pathological significance of RAB5 hyperactivation, we examined endosomal Rabs and lysosomal enzymes in DS with and without AD, as well as in the Dp16 mouse model of DS. The Dp16 mouse is trisomic for mouse genes that are homologous to those on human chromosome 21 (Hsa21), including *App*, and exhibits features relevant to DS‐AD pathogenesis.^25^, ^26^, ^27^ Our results showed widespread hyperactivation of endosomal Rabs together, along with increased levels of lysosomal cathepsins B and L. Our findings revealed that increased activation of RABs 7 and 11 was associated with upregulation of their GEFs, as well as enhancing binding RABs 7 and 11 and their GEFs to endosomal membranes. Of note, hyperactivation of endosomal Rabs and the upregulation of GEFs for RABs 7 and 11 directly depended on increased *App* gene dose. Our findings reveal that increased *App* gene expression acts through RAB5 hyperactivation to cause ELN dysregulation. They suggest a novel mechanism in which APP β‐CTF supports persistent RAB5 activation, disrupting the endosomal Rab cascade to compromise EE maturation with dysregulation of downstream endolysosomal compartments. These results motivate the potential for developing *APP‐* and/or *Rab5*‐targeted treatments to address endolysosomal pathologies in DS and related conditions.

## 2 METHODS

### 2.1 Ethics approval

All the procedures related to human samples were carried out under a protocol reviewed and approved by the human subjects review board at the University of California San Diego (UCSD; institutional review board [IRB] #: 180620). All the animal studies were performed strictly according to the recommendations in the Guide for the Care and Use of Laboratory Animals of the National Institutes of Health. All animal experiments have been approved by the University of California San Diego Institutional Animal Care and Use Committee (Protocol #: S09315).

### 2.2 Reagents

GTP–Agarose (G9768), Isopropyl β‐d‐thiogalactoside (15502), Halt Protease and Phosphatase Inhibitor Cocktail (78440), Cycloheximide (CHX; C4859), and Poly‐D‐Lysine Hydrobromide (PDL; P6407) were obtained from MilliporeSigma. Glutathione Sepharose 4B (17075601) was from GE Healthcare. Hanks' Balanced Salt Solution (HBSS, 14185052), Trypsin (2.5%, 10×) (15090046), Neurobasal Medium (21103049), B‐27 Supplement (17504044), GlutaMAX Supplement (35050061), and DQ Red BSA (DQ‐BSA; D12051) were from Thermo Fisher Scientific. DNase I (10104159001) was from Roche. Fetal bovine serum (FBS; FB‐02) was from Omega Scientific. DexoMAG iron‐dextran (DexoMAG 40) was from Liquids Research. BMV109 was a gift from Dr. Matthew Bogyo (Stanford University School of Medicine). Protein A/G PLUS‐Agarose beads (sc‐2002) were from Santa Cruz Biotechnology. The Iscript cDNA Synthesis Kit (1708891) was from Bio‐Rad. The Quick‐RNA Miniprep Kit (R1054) was from Zymo Research. Cathepsin B Activity Assay Kit (ab65300), Cathepsin L Activity Assay Kit (ab65306), and Cathepsin D Activity Assay Kit (ab65302) were from Abcam. *Escherichia coli* strain BL21 (C2530H) was from New England Biolabs.

**RESEARCH IN CONTEXT**

1. **Systematic review**: It is well‐established that in Down syndrome (DS), early endosome dysfunction is associated with increased RAB5 activity. However, the extent of RAB5 hyperactivation's impact on the dysregulation of downstream endosomal Rabs remains unclear. In addition, there is limited insight into the mechanisms driving RAB5 hyperactivation and its effects across both DS brain tissue and DS mouse models.
2. **Interpretation**: Our findings reveal a novel mechanism whereby amyloid precursor protein (APP) beta‐C‐terminal fragment (β‐CTF) sustains RAB5 activation, disrupting the endosomal Rab cascade and impairing early endosome maturation, leading to dysregulation of downstream endolysosomal compartments.
3. **Future directions**: These findings highlight the potential to develop therapies targeting *APP* and/or *Rab5* to address endolysosomal dysfunction in DS and related disorders.

### 2.3 Constructs

The Lox‐Syn lentivirus vector was used to produce lenti‐β‐CTF, lenti‐α‐CTF, lenti‐RAB5^S34N^, and lenti‐RAB5^Q79L^ in neurons. This vector contains two distinct neuronal‐specific synapsin promoters, one regulating the expression of the specified proteins and the other controlling green fluorescent protein (GFP) expression.^28^ The corresponding control vector was created by deleting DsRed while retaining GFP in Lox‐Syn. Annealed oligonucleotides targeting mouse *Appl1* mRNA were cloned into the lentiviral vector pLKO.1 (Addgene plasmid #8453). Luciferase‐targeting short hairpin RNA (shRNA) (shLuc) was used as the negative control. GFP‐RAB5, mCherry‐RAB7, DsRed‐RAB11, and mCherry‐RAB4 were subcloned into pLenti CMV GFP Puro (Addgene plasmid #17448). GFP‐RAB5 was kindly provided by Dr. Marino Zerial (Max Planck Institute of Molecular Cell Biology and Genetics). mCherry‐RAB7 was obtained from Dr. Chengbiao Wu (UCSD). mCherry‐RAB4 (Addgene plasmid #55125) and DsRed‐RAB11 (Addgene plasmid #12679) were sourced from Addgene. The pGEX‐4T‐1 vector was a gift from Dr. Junbao Fan (UCSD). The pGEX‐4T‐2/Rabaptin‐5: R5BD vector was provided by Dr. Guangpu Li (University of Oklahoma Health Sciences Center). The pGEX‐4T‐3/RILP: R7BD vector (Addgene plasmid #79149) was acquired from Addgene. The pGEX‐6P/MICAL‐L2: R10BD vector was a gift from Dr. Gustav Lienhard (Geisel School of Medicine at Dartmouth). The pMD2.G (Addgene plasmid #12259) and psPAX2 (Addgene plasmid #12260) vectors were also sourced from Addgene.

### 2.4 Human *post‐mortem* brain samples

Frozen human DS, DS‐AD, and control frontal cortices (male and female) (DS, *n* = 12, age 22–62; DS‐AD, *n* = 18, age 41–65; control for DS (C/DS), *n* = 12, age 48–59; and control for AD‐DS (C/DS‐AD), *n* = 18, age 39–59) were stored at −80°C and obtained from National Institutes of Health (NIH) NeuroBioBank and University of California Irvine Institute for Memory Impairments and Neurological Disorders (Tables S1–S3). Human AD cortices (AD, *n* = 14, age 64–100; control for AD (C/AD), *n* = 11, age 58–103) were received from University of California San Diego Shiley‐Marcos Alzheimer's Disease Research Center and Banner Sun Health Research Institute (Tables S1 and S4). In addition, one rare case of DS with partial trisomy 21(PT), as previously reported,^29^ was included in this study (Table S2). The cognitive status of individuals with DS‐AD and AD was documented for the samples obtained from aforementioned sources.

### 2.5 Mice

Dp (16)1Yey/+ (Dp16; JAX:013530; The Jackson Laboratory) mice harbor a duplication orthologous to the human chromosome 21q11‐q22.3 and carry 113 genes homologous to those on HSA21. To maintain the Dp16 mice, females were crossed with (C57BL/6J [JAX:000664; The Jackson Laboratory] × C3H/HeJ [JAX:000659; The Jackson Laboratory] F1 mice (B6C3)). Diploid (2N) littermate mice, with identical genetic backgrounds, were utilized as controls. All other mice in this study were male unless otherwise specified. *App*−/− mice in which *App* was inactivated by deleting the *App* promoter and its first exon^30^ were crossed individually with C57BL/6J and C3H/HeJ mice. The resulting offspring were then intercrossed to produce F1 mice (B6C3: *App *+/‐). To generate Dp16 mice with only two mouse *App* alleles (i.e., Dp16: *App*+/+/−), Dp16 mice were bred with B6C3: *App*+/− mice on the same strain background. The animals' genotype was confirmed via polymerase chain reaction (PCR) using tail samples for genomic DNA extraction. The PCR protocol included amplification of the *HPRT* insertion specific to Dp16 mice, alongside amplification of the *Il‐2* gene serving as an internal control. Amplification of the *mApp* fragment and the *Neo* insertion was used to screen for *App* allele deletion. The primers utilized are listed in Table S5. All animals were bred and maintained following standard procedures, housed two to five per cage with a 12‐h light–dark cycle, and provided ad libitum access to food and water. C57BL/6J mice were crossed to produce wild‐type (WT) embryos. To generate *App−*/− embryos, B6C3: *App*+/‐ mice were crossed to produce *App−*/− offspring. Subsequently, these *App−*/− offspring were bred with each other to yield *App−*/− embryos.

### 2.6 GTP agarose pull‐down assay

The levels of GTP‐bound Rabs were quantitated following published methods. Briefly, primary 2N or Dp16 cortical neurons (3 × 10^6^ neurons in a 60 mm dish) treated as indicated were collected in lysis buffer (50 mM Tris–HCl pH 7.5, 250 mM NaCl, 5 mM Mg‐Acetate, 0.5% Triton X‐100, protease inhibitor cocktail, and 0.2 mM sodium orthovanadate) and rotated at 4°C for 30 min. Dissected mouse brain tissues or frozen post‐mortem human frontal cortices (Table S6) were homogenized in the same buffer using 10–15 strokes in a 2‐mL WHEATON Dounce homogenizer (DWK Life Sciences) and then rotated at 4°C for 30 min. All lysates were centrifuged (16,000 *g* for 15 min at 4°C) to produce supernatants. The protein content in supernatants from mouse and human tissues was quantified using the Bradford assay. An aliquot of supernatants was saved to measure the total levels of individual Rabs. Equal amounts of supernatants were incubated with 100–150 µL GTP‐agarose beads overnight at 4°C with rotation. For brain tissues, 1 mg of total protein in lysate for each sample was used. The beads were washed in the same lysis buffer but without the protease inhibitors three times (5 min/each, 4°C) and then boiled in sodium dodecyl sulfate–polyacrylamide gel electrophoresis (SDS‐PAGE) sample buffer. The amounts of GTP‐Rabs were measured by western blotting.

### 2.7 GST pull‐downs

GST‐Rabaptin‐5, Rab interacting lysosomal protein (RILP), or MICAL like 2 (MICAL‐L2) was introduced into *E. coli* strain BL21. One hundred milliliters of Luria Broth was inoculated with 0.5 mL of overnight culture and incubated at 37°C until reaching an optical density (OD) of 0.6–0.8. Isopropyl β‐d‐thiogalactoside was added to a final concentration of 0.5 mM to initiate protein production. The 100 mL culture was further incubated for 2–3 h at 37°C. Subsequently, the bacteria were harvested by centrifugation, washed with cold phosphate‐buffered saline (PBS), and resuspended in 5 mL of cold lysis buffer (25 mM Tris–HCl pH 7.4, 1 M NaCl, 0.5 mM EDTA, 1 mM DTT, 0.1% Triton X‐100, and protease inhibitor cocktail), followed by sonication. The bacterial lysates were then cleared by centrifugation, and 5 mL of cold lysis buffer was added. Proteins were purified by incubating 250 µL of a pre‐equilibrated 50% slurry of glutathione sepharose 4B beads with the lysate for 30 min at room temperature, followed by washing with lysis buffer and resuspension as a 50% slurry. Protein quantification was conducted using the Bradford assay. For the pull‐down assays, 4‐month‐old 2N and Dp16 mouse half‐brains were lysed in pull‐down buffer (20 mM HEPES, 100 mM NaCl, 5 mM MgCl_2_, 1% Triton X‐100, and protease inhibitors). Each pull‐down reaction was carried out in 1 mL of solution containing 150 µg of cell lysate and 15 µg of beads pre‐equilibrated in pull‐down buffer. The reaction mixtures were then rocked overnight at 4°C, washed twice with cold pull‐down buffer, and the bound proteins were eluted by SDS‐PAGE sample buffer at 95°C for 10 min.

### 2.8 RNA isolation and quantitative PCR

Total RNA was extracted from the cortex or hippocampus of 2N and Dp16 mice or frontal cortices from DS‐AD and C/DS‐AD using a Quick‐RNA Miniprep Kit. Equal amounts of total RNA were utilized for cDNA synthesis with the iScript cDNA Synthesis Kit, following the manufacturer's instructions. The primer sequences are provided below (Table S5), with many obtained from PrimerBank.^31^ qPCR was conducted for 40 cycles. Values within the log‐linear phase of the amplification curve were determined for each probe/primer set and analyzed using the ΔΔCt method (Applied Biosystems 7300 Real‐Time PCR System).

### 2.9 Primary neuron culture

E18 WT mouse embryos and 2N and Dp16 embryos from Dp16 mice bred as described above were used to generate cortical neuron cultures following established protocols.^23^ Genomic DNA for genotyping was extracted from tail samples taken from each embryo, using the same procedure described above. Cortical tissue was dissected from the embryos and finely minced using a sterile, fire‐polished Pasteur glass pipette, followed by gentle trituration. The tissue was further dissociated with 1× trypsin in HBSS for 5 min at 37°C in a water bath, followed by DNase I treatment (1 mg/mL). Subsequently, the tissue was dissociated into single cells through additional trituration. The dissociated cells were neutralized using Neurobasal media with 10% FBS, B‐27, and GlutaMAX, and then briefly centrifuged at 233 *g* for 5 min. The pelleted neurons were resuspended and plated in Neurobasal medium supplemented with 10% FBS, B‐27, and GlutaMAX in PDL‐coated dishes and incubated overnight. The next day, the media were replaced with maintenance medium (Neurobasal with B‐27 and GlutaMAX only). Half of the media were refreshed every 2–3 days until the experiments were conducted.

### 2.10 Fluorescence‐tagged Rabs live imaging

Primary neurons from mouse strains 2N and Dp16 were cultured in vitro for 2 weeks. These neurons underwent co‐infection with pLenti CMV GFP Puro‐GFP‐RAB5 and distinct lentiviruses containing RAB4, RAB7, or RAB11. Following a 3‐day infection period, live cell imaging was performed to evaluate the Rab proteins' subcellular localization, employing a Leica TCS SPE confocal microscope.

### 2.11 Protein degradation assays

Mass cultures of either 2N or Dp16 cortical neurons (3.5 × 10^5^ neurons in each well of a 24‐well plate) at days in vitro (DIV)8 were treated with 100 µg/mL CHX for the indicated durations. Neurons were briefly washed with cold PBS and then harvested and lysed in PBS containing 1% NP‐40, 0.1% SDS, 0.1% sodium deoxycholate, and Halt Protease and Phosphatase Inhibitor Cocktail. All lysates were centrifuged to produce supernatants, which were then boiled in an SDS‐PAGE sample buffer.

### 2.12 Lentivirus production and transduction

Lentiviral particles were produced in HEK293T cells by co‐transfecting them with expression vectors and the viral packaging plasmids pMD2.G and psPAX2 using the calcium phosphate transfection method. The viruses were concentrated by ultracentrifugation (24,000 rpm, 2 h, SW‐28 rotor), resuspended in PBS (Ca^2+^/Mg^2+^‐free), and added to neurons.

### 2.13 Lysosome magnetic isolation

Lysosomes were isolated magnetically following a previously established protocol with slight adjustments. Neurons were seeded onto PDL‐coated 10‐cm culture dishes at a density of 10 × 10^6^ cells per dish. One day before isolation, 1 mL of DexoMAG iron‐dextran solution (10 mg/mL) was added to 10 mL of neuronal culture media and incubated for 20 h. Subsequently, cells were washed twice with cold PBS, scraped, and pelleted by centrifugation at 300 × *g* for 5 min at 4°C. The pellet was lysed using a 25 G needle in cold SCA buffer (20 mM HEPES‐KOH pH 7.5, 10 mM KCl, 1.5 mM MgCl_2_, 1 mM EDTA, 1 mM EGTA, and 250 mM sucrose supplemented with Halt Protease and Phosphatase Inhibitors). The ruptured cells were centrifuged at 800 × *g* for 5 min at 4°C, and the supernatant was collected. This process was repeated once, and the combined supernatant containing cytosol and organelles was designated as the postnuclear supernatant (PNS). The PNS was then loaded onto an LD Column (Miltenyi Biotec) attached to a QuadroMACS Separator magnet, which had been pre‐equilibrated with 1 mL of PBS containing 0.5% BSA, and washed with 1 mL of PBS. After loading, the column was washed successively with SCA buffer and PBS. Finally, lysosome fractions (lyso‐frs) were eluted by detaching the column from the magnet and plunging with 200 µL of elution buffer (PBS, 0.5 mM sucrose, and Halt Protease and Phosphatase Inhibitors). All isolation steps were carried out at 4°C.

### 2.14 Cathepsin activity measurement with BMV‐109

Mouse cortical neurons were plated on PDL‐coated 12‐well dishes and cultured for 2 weeks. BMV‐109, a pan‐cysteine cathepsin activity‐based probe that enables simultaneous monitoring of the activities of cathepsins X, B, S, and L,^32^ was diluted in neuronal culture medium to a final concentration of 1 µM and incubated with cells at 37°C for 45 min. After this incubation period, the culture media were gently aspirated, and the cells were washed with PBS. Cells were then harvested and lysed with 1× SDS loading buffer. The resulting lysates were loaded onto an SDS‐PAGE gel along with a fluorescent molecular weight marker. The gel was scanned using the Typhoon Imager, selecting the excitation and emission parameters appropriate for the Cy5 dye. After scanning, the gel was transferred to a polyvinylidene difluoride (PVDF) membrane for western blotting analysis.

### 2.15 In vivo measurement of cathepsin activity

Cathepsin activities were assessed using the cathepsin B, L, and D activity assay kits following the manufacturer's instructions. In brief, half‐brain tissue (∼200 mg) freshly harvested was washed with cold PBS and homogenized with 1 mL of lysis buffer on ice. The samples were centrifuged at 16,000 × *g* for 5 min at 4°C to remove insoluble material, and the resulting supernatant was collected. This supernatant was incubated with the respective substrate at 37°C for 1 h. Following incubation, the samples were analyzed using Varioskan LUX Multimode Microplate Reader (Thermo Fisher Scientific). Cathepsin activity was determined by normalizing it to the protein content in the lysate.

### 2.16 Lysosome degradation activity assay in neurons

DQ‐BSA is a fluorescent probe composed of a BSA derivative conjugated to the self‐quenched red‐BODIPY dye. It generates brightly fluorescent puncta upon lysosomal degradation, allowing assessment of proteolytic activity within lysosomal compartments. Two‐week‐old 2N and Dp16 mouse cortical neurons or WT neurons infected with lenti‐RAB5^Q79L^ for 48 h were treated with 10 µg/mL of DQ‐BSA diluted in the culture medium for 6 h. Subsequently, cells were washed once with HBSS solution and switched to a fresh pre‐warmed medium. Live cell images were captured immediately following the treatment. The fluorescence intensity of DQ‐BSA staining in the soma was quantified using ImageJ software. Specifically, the Corrected Total Cell Fluorescence (CTCF) values were determined by measuring the fluorescence intensity in the soma of each cell and then correcting for background fluorescence. The 2N and Dp16 neurons under the same setting were also processed for western blotting to detect internalized DQ‐BSA using a BSA antibody.

### 2.17 Endosome purification and co‐immunoprecipitation assay

Brains from 9‐ to 10‐month‐old Dp16 and 2N mice were treated with 1 mL of pre‐cooled homogenization buffer (10 mM HEPES pH 7.4, 1 mM EDTA, 0.32 M sucrose, and Halt Protease and Phosphatase Inhibitors) and homogenized by stroking 20 times on ice using a 2 mL Dounce homogenizer. The lysates were then centrifuged at 800 × *g* for 10 min, and the supernatant was collected. The pellet was resuspended in another 1 mL homogenization buffer with five gentle strokes and centrifuged again at 800 × *g* for 10 min. The combined supernatants from the first and second centrifugations were further centrifuged at 3500 × *g* for 10 min and then at 23,000 × *g* for 20 min. The resulting pellet was suspended in incubation buffer containing 2 mM EDTA and 5% BSA in PBS and subjected to immuno‐isolation with agarose beads. Protein A/G PLUS‐Agarose beads (sc‐2002; Santa Cruz Biotechnology) were washed twice with wash buffer (PBS containing 0.1% BSA) for 5 min each at 4°C with rotation, and then incubated for 5 h in blocking buffer (0.2 M Tris–HCl pH 8.5, and 0.1% BSA) at room temperature. After the agarose beads were washed, they were incubated in PBS containing 0.1% BSA with either 1 µg anti‐RAB5 antibody (Abcam, ab218624 or Synaptic Systems, 108111) or rabbit IgG or mouse IgG control antibody overnight at 4°C with rotation. Following incubation, the beads were washed four times (5 min each) at 4°C and then mixed with purified light membrane lysates, followed by incubation for 2 h at 4°C. Subsequently, the beads were washed five times with incubation buffer and twice with PBS for 10 min each time. Finally, the beads were resuspended in 50 µL of 1× SDS loading buffer for western blot analysis.

### 2.18 Human sample processing

Human samples of ≈10–20 mg frozen tissue were processed in 0.5 mL RIPA buffer (50 mM Tris–HCl pH 8.0, 150 mM NaCl, 1% NP‐40, 1% Triton X‐100, 0.1% sodium deoxycholate, 0.1% SDS, and protease inhibitor cocktail) and rotated in 4°C for 30 min. All lysates were bench centrifuged (16,000 *g* for 15 min at 4°C) to produce supernatants, and the protein content of supernatants of each sample was determined using the Bradford assay. Samples were examined using western blotting analysis.

### 2.19 Western blotting

Equal total proteins for each sample (10–20 µg) were separated on SDS‐PAGE and then electrotransferred to PVDF membranes. The membranes were blocked with 5% nonfat milk for 1 h and probed with specific primary antibodies (Table S7) overnight at 4°C followed by incubation with goat anti‐rabbit IgG‐HRP (1:15,000) or anti‐mouse IgG‐HRP (1:15,000) at room temperature for 1 h. All blots were developed using the BioRad Clarity Western ECL substrate and captured using ChemiDoc XRS + (Bio‐Rad); only blots within signals in the linear range were quantitated using the ImageLab 3.0.1 software (Bio‐Rad).

### 2.20 Statistic analysis

All data are presented as the mean ± standard error of the mean (SEM). Statistical analyses were performed using PRISM with a two‐tailed Mann–Whitney *U* test (human samples), Student *t*‐test, or one‐way analysis of variance (ANOVA) followed by the Newman–Keuls Multiple Comparison test. The significance levels were *p < 0.05, **p < 0.01, and ***p < 0.001.

## 3 RESULTS

### 3.1 Hyperactivation of endosomal Rabs in DS‐AD and DS frontal cortex

**FIGURE 1:**
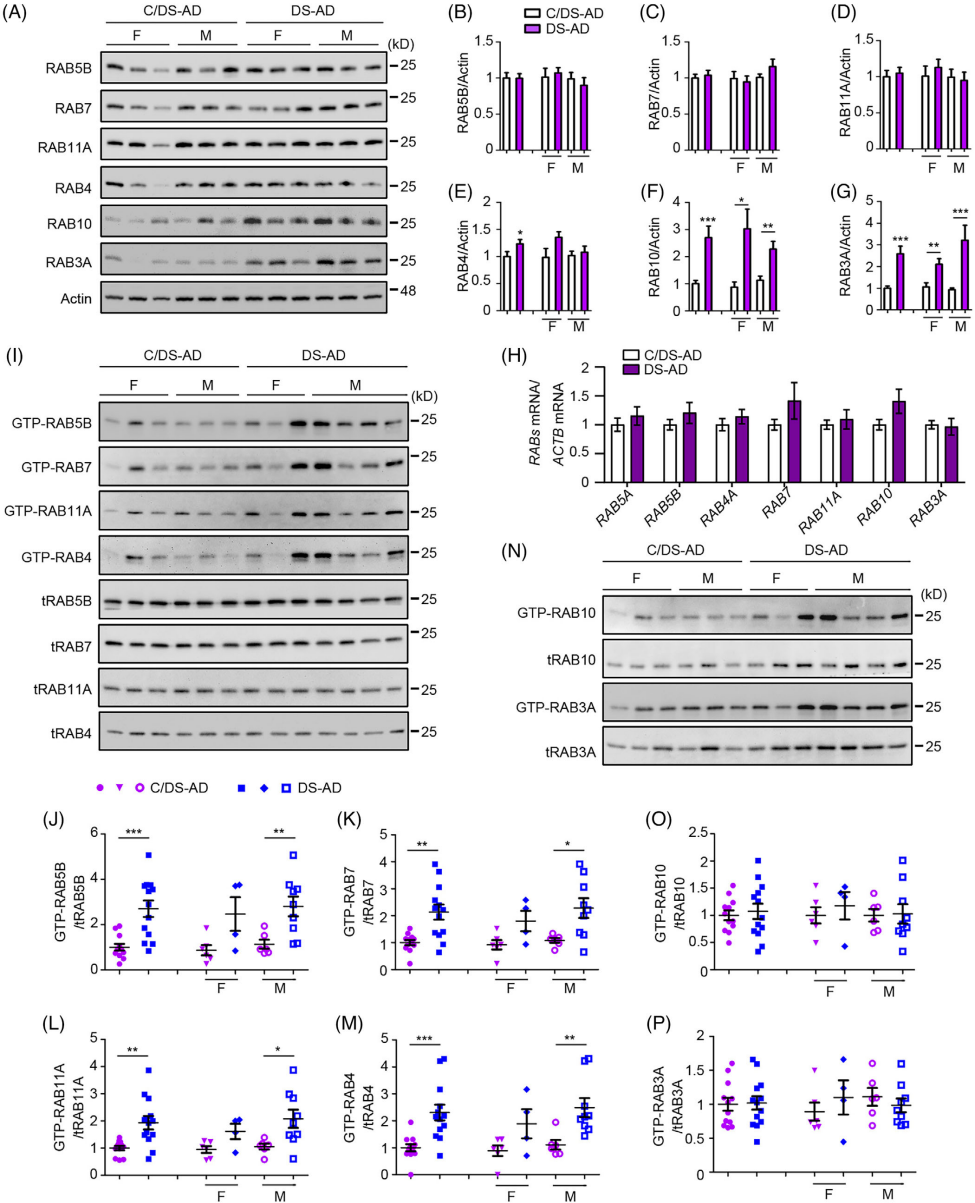
Widespread hyperactivation of endosomal Rabs in DS‐AD frontal cortex. (A) Western blot analysis of the levels of different Rabs in protein extracts from the frontal cortex of patients with DS‐AD and C/DS‐AD. β‐Actin was used as a loading control. (B–G) Quantitative analysis and statistical comparison of Rab levels in combined, female, and male samples from DS‐AD and C/DS‐AD groups. (H) The relative mRNA levels of different Rabs in the frontal cortex of DS‐AD and C/DS‐AD were assessed by qPCR. *ACTB* mRNA was used as an internal control. (I) GTP agarose pull‐down assay was used to measure the activities of RABs 5, 4, 7, and 11 in the frontal cortex of DS‐AD and C/DS‐AD. (J–M) Quantitative analysis and statistical comparison of the activities of different Rabs in DS‐AD and C/DS‐AD groups. (N) The activities of RABs 10 and 3 were measured in the frontal cortex of DS‐AD and C/DS‐AD. (O, P) Quantitative analysis and statistical comparison of the activities of RABs 10 and 3 in DS‐AD and control groups. Mann–Whitney *U* test; F, female; M, male; *n* = 18 for C/DS‐AD (F: 9, M: 9), *n* = 16 for DS‐AD (F: 9, M: 7) for A to G; *n* = 10 for C/DS‐AD, *n* = 8 for DS‐AD for H; *n* = 12 for C/DS‐AD (F: 6, M: 6), *n* = 13 for DS‐AD (F: 4; M: 9) in I to P; *p < 0.05, **p < 0.01, ***p < 0.001. AD, Alzheimer's disease; C/, control for; DS, Down syndrome; mRNA, messenger RNA; qPCR, quantitative polymerase chain reaction.

Previous studies have reported EE enlargement and increased RABs 4, 5, and 7 proteins in DS fibroblasts.^33^ Herein we undertook the first comprehensive evaluation of endosomal Rabs in brains of individuals with DS with and without AD (DS‐AD and DS). There were no differences in RABs 5, 7, or 11 levels in the frontal cortex of individuals with DS‐AD compared to controls. However, RAB4 levels were slightly increased in DS‐AD (Figure 1A–E). We also examined RAB10, which mediates endosomal membrane trafficking and has functional overlap with RAB5,^34^ and RAB3, which regulates neurotransmitter release and synaptic plasticity.^35^ Both these Rabs have been implicated in AD^36^, ^37^ but have not been explored in DS. Unexpectedly, RAB3 and RAB10 levels were significantly elevated in both sexes with DS‐AD (Figure 1A,F,G). The increases in RABs 3 and 10 were not accompanied by significant differences in messenger RNA (mRNA) levels; nor were there significant changes in the mRNAs for RABs 5, 7, 11, or 4 (Figure 1H). As for DS‐AD, in DS brain there were increases in RABs 4, 3, and 10, but not in RABs 5, 7, or 11 (Figure S1A–G).

Although RAB5 marks enlarged early endosomes in DS fetuses^38^ and DS mouse models,^39^, ^40^ and its hyperactivation has been shown in DS models,^23^, ^41^ RAB5 activity has not been directly explored in DS brains. Our study found increased total and specific activity (GTP‐loading) of RAB5B in the frontal cortex tissues of DS‐AD compared to age‐matched cognitively normal controls, particularly in males (Figure 1I,J). Rab cascades regulate EE maturation into RE and LE compartments.^8^ The activities of endosomal Rabs downstream of RAB5 had not been investigated previously in DS. It is striking that significant increases in the total and specific activity of RABs 4, 7, and 11 were detected in DS‐AD brains (Figure 1I,K–M). Although increased, the total activity of RAB3 and RAB10 was not different from controls when normalized to protein levels (Figure 1N–P). Similarly, individuals with DS exhibited increased total activities of RABs 4, 5, 7, and 11A (Figure S1H–L). Extending the analysis to the AD frontal cortex in comparison to healthy controls, as in DS and DS‐AD, we found no significant differences in the levels of RABs 5, 7, and 11A; distinct from DS and DS‐AD, no increases were detected for RABs 4, 3, or 10 (Figure S2). The findings are evidence of widespread hyperactivation of endosomal Rabs in DS brains.

### 3.2 Imbalances in endosomal Rab GEFs/GAPs in DS‐AD and DS

**FIGURE 2:**
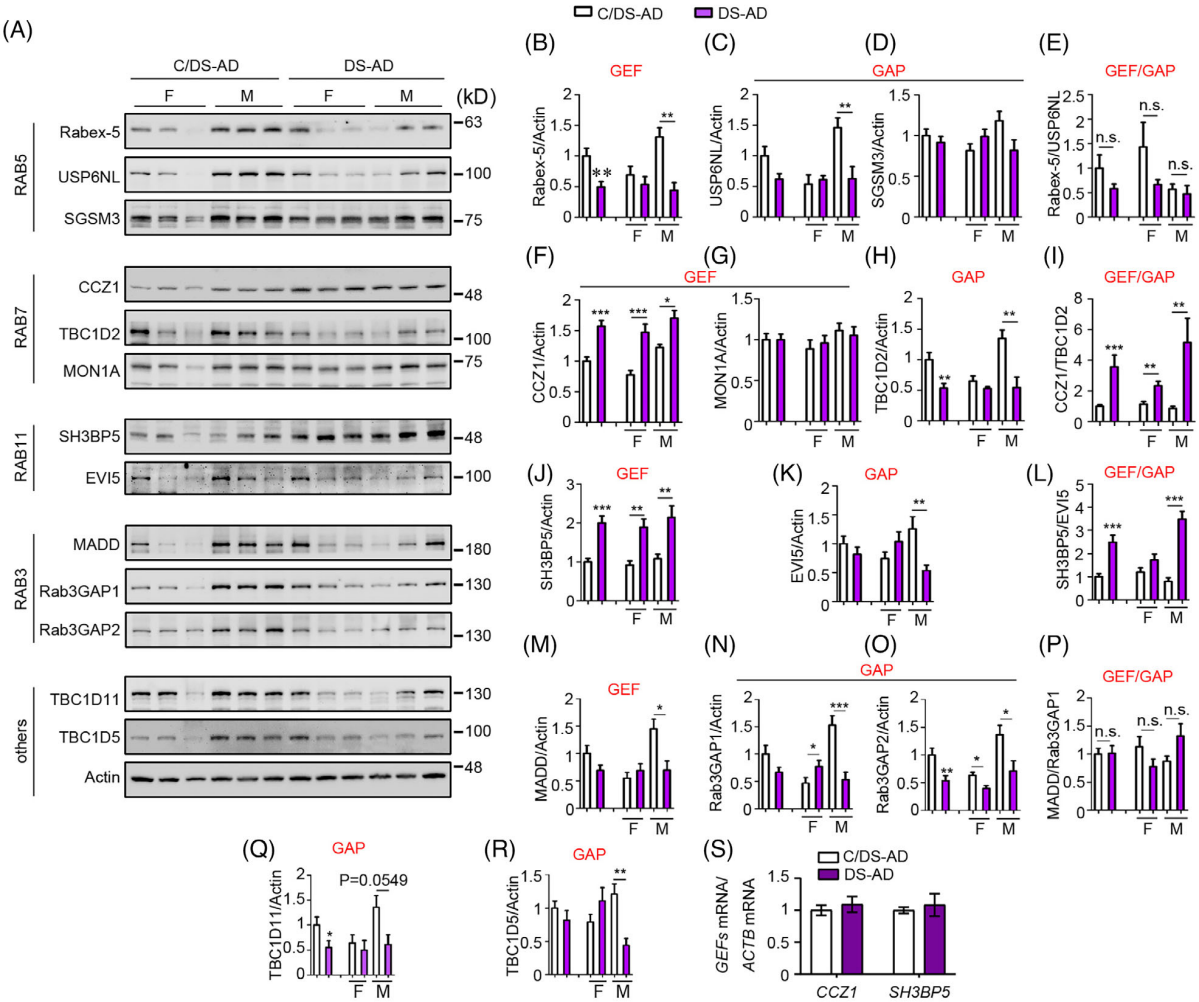
Selective alterations of the GEFs of endosomal Rabs in DS‐AD frontal cortex. The levels of GEFs/GAPs of RAB5 (A–E), RAB7 (A, F–I), RAB11 (A, J–L), and RAB3 (A, M–P) were measured in the frontal cortex of DS‐AD and C/DS‐AD. Quantitation and statistical analysis were displayed on the right panels. (Q, R) The levels of other GAPs TBC1D11 and TBC1D5 were also analyzed in these samples. (S) The mRNA levels of *CCZ1* and *SH3BP5* in the frontal cortex of DS‐AD and C/DS‐AD were assessed by qPCR. Mann–Whitney *U* test; F, female; M, male; *n* = 18 for C/DS‐AD (F: 9, M: 9), *n* = 16 for DS‐AD (F: 9, M: 7) for A to R; *n* = 10 for C/DS‐AD, *n* = 8 for DS‐AD for S; *p < 0.05, **p < 0.01, ***p < 0.001. AD, Alzheimer's disease; C/, control for; DS, Down syndrome; GAP, GTPase‐activating protein; GEF, guanine‐nucleotide exchange factor; mRNA, messengaer RNA, qPCR, quantitative polymerase chain reaction.

Maturation and trafficking of endosomal compartments are regulated by specific GEFs that recruit and activate their cognate Rabs^42^ and the coordinated activity of specific GAPs. We investigated if differences in endosomal Rab activity correlated with changes in their GEFs and GAPs or the ratio of GEF/GAP levels^9^ in DS‐AD and DS. Rabex‐5 (a RAB5 GEF) and USP6NL (a RAB5 GAP), both increased in male controls, but with unchanged Rabex‐5/USP6NL ratios. No significant differences were found for SGSM3 (another RAB5 GAP) (Figure 2A,B–E). The CCZ1‐MON1 complex^43^ and SH3BP5^44^ are GEFs for RABs 7 and 11, respectively. CCZ1 levels were significantly higher in DS‐AD males and females, whereas MON1A levels remained unchanged. The levels of RAB7 GAP, TBC1D2,^45^ were higher in male controls compared to males with DS‐AD, resulting in a significantly elevated CCZ1/TBC1D2 ratio in DS‐AD patients (Figure 2A,F–I). Similarly, SH3BP5 levels were notably higher in DS‐AD and the levels of RAB11 GAP, EVI5,^46^ were higher in male controls, leading to increased SH3BP5/EVI5 ratios, especially in males (Figure 2A,J–L). The levels of RAB3 GEF (MADD) and GAPs (Rab3GAP1 and Rab3GAP2)^9^ were significantly elevated in male controls, with no significant differences in the MADD/Rab3GAP1 ratio between DS‐AD and controls (Figure 2A,M–P). Additional RABs 7 and 11 GAPs (TBC1D11, TBC1D5)^9^ were also increased in male controls relative to males with DS‐AD (Figure 2A,Q,R). The observed increases in CCZ1 and SH3BP5 were not reflected in their respective mRNA levels (Figure 2S). Findings in DS brain tissues largely mirrored those in DS‐AD, including increased GEF/GAP ratios for RABs 7 and 11 and comparable ratios for RABs 5 and 3 compared with those in controls (Figure S3).

Increased *APP* gene dose and elevated β‐CTF levels drive EE enlargement and RAB5 hyperactivation.^15^, ^16^, ^17^ Notably, a patient with PT, who had only two copies of the *APP* gene and showed no evidence of cognitive decline in late life or AD pathology at post‐mortem exam,^29^ showed Rab activities and CCZ1 and SH3BP5 levels comparable to cognitively normal controls (Figure S4), supporting a role for increased *APP* gene dose involvement in observed changes.

### 3.3 Increased lysosomal cathepsin levels in DS‐AD and DS

Lysosomes are functionally linked to endosomes, and their dysfunction has been implicated in the pathogenesis of neurodegenerative diseases,^11^ including AD and DS. Because lysosomal cathepsins B, L, and D are linked to AD,^47^, ^48^, ^49^ we investigated their levels in the frontal cortex of individuals with DS‐AD and DS. Cathepsins B and L showed significant increases in the absence of changes in their mRNAs (Figure S5A–I). As reported earlier,^50^ the mature form of cathepsin D, the major form in humans, was unchanged (Figure S5D,H). We also assessed the transcription factors associated with cathepsin expression (TFE3, TFEB, pS211‐TFEB). TFE3 and TFEB showed no significant changes, although TFEB exhibited a trend toward lower levels. Although levels of pS211‐TFEB were reduced in DS‐AD, this change was not significant when normalized to TFEB (Figure S5J,K). Moreover, although we observed increases in cathepsins B and L in the PT brain tissue compared to controls, the levels detected were less than the average levels typically detected in the brains of patients with DS‐AD (Figure S5L–N). Taken together, our findings are evidence for marked dysregulation of the endolysosomal pathway in DS‐AD and DS and support a link between increased *APP* gene dose and the changes detected.

### 3.4 Hyperactivation of endosomal Rabs in the brains of Dp16 mice

To explore the cellular and molecular basis for dysregulation of the endolysosomal system in DS and DS‐AD we asked if the Dp16 mouse model of DS recapitulated the changes observed in DS brains. The Dp16 mouse model harbors three copies of ≈115 genes from mouse chromosome 16, homologous to those encoded on the long arm of Hsa21, including three copies of the *App* gene. Forebrain tissues from both young (4 months) and aged (16 months) Dp16 mice exhibited increased activity of RABs 5B, 4, 7, and 11A compared to 2N (euploid) controls, with unchanged protein levels (Figure S6A–F), indicating increased total and specific endosomal Rab activity. Increased *App* gene dose corresponded to an ≈50% increase in full‐length APP (fl‐APP) (Figure S6A,D,G,H). Unlike DS brains, there was a significant increase in total and specific RAB10 activity in the 4‐month‐old Dp16 forebrain; no differences were found in RAB3 activity (Figure S6I–M). As in the DS‐AD brain, no differences were detected in mRNA levels encoding these Rabs between 4‐month‐old 2N and Dp16 mice (Figure S6N–T). The findings are evidence that the widespread hyperactivation of endosomal Rabs in DS brains is recapitulated in both the young adult and aged Dp16 brain via demonstration of increased total and specific activities of RABs 4, 5, 7, and 11.

The three isoforms of RAB5 (RABs 5A, 5B, and 5C) share over 90% sequence identity.^51^ Hyperactivity of RAB5A and RAB5C was also observed in 4‐month‐old Dp16 brain tissues (Figure S7A,B). Regional differences were noted across the cortex, hippocampus, septum, cerebellum, and striatum, with significant RAB5 activity increases in all brain regions except the striatum of 7‐ to 8‐month‐old Dp16 mice (Figure S7C–G). Finally, the findings of increased fl‐APP and RAB5 activity in males were replicated in the cortex of 7‐ to 8‐month‐old female Dp16 mice (Figure S7H,I).

### 3.5 Increased binding of endosomal Rab effectors with Rabs in Dp16 mice

Active Rab proteins interact with specific effectors to regulate cellular functions.^52^ Pull‐down assays were used to measure Rab protein binding to glutathione‐S‐transferase (GST)–fused fragments of specific effector proteins to explore the impact of increased endosomal Rab activity. Our results revealed that GTP‐bound RABs 5, 7, and 10 interacted, respectively, with Rabaptin‐5,^53^ RILP,^54^ and MICAL‐L2.^55^ Increased levels of the effectors associated with each of these Rabs were detected in the forebrains of 4‐month‐old Dp16 mice (Figure S8A–C), confirming that RABs 5, 7, and 10 hyperactivation is linked to increased binding to their specific effectors.

### 3.6 *App* gene dose‐dependent hyperactivation of endosomal Rabs in Dp16 mice

**FIGURE 3:**
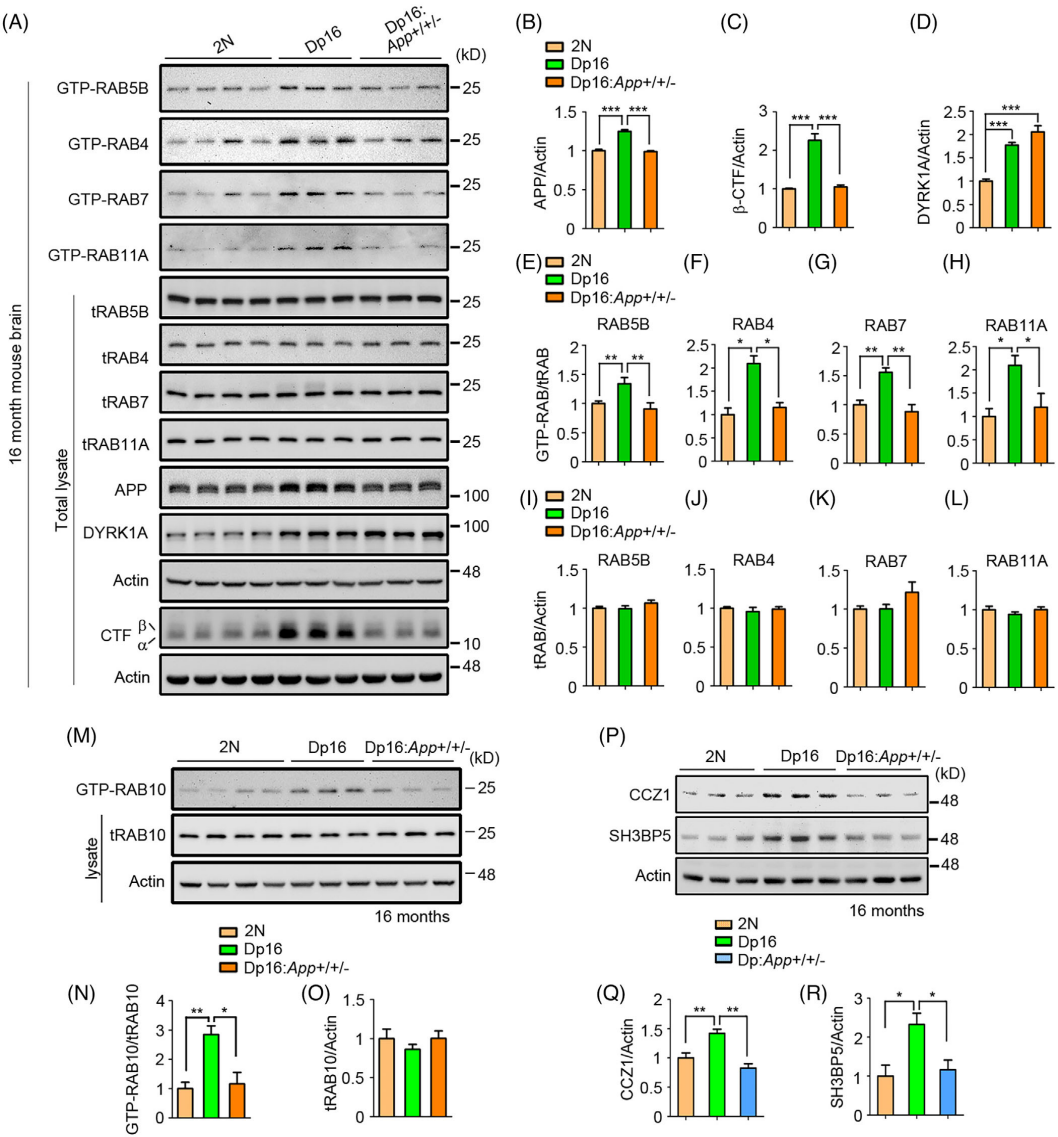
APP‐dependent endosomal Rab hyperactivation and GEF increase in Dp16 brains. (A) The levels of GTP‐loaded RABs 5, 4, 7, and 11, and total Rabs were assayed in brain homogenates from 16‐month‐old 2N, Dp16, and Dp16: *App+/+/‐ * mice. (B–D) Quantitation and statistical analysis of the levels of APP, β‐CTF, and DYRK1A in the same mice. (E–L) Quantitation and statistical analysis of the GTP‐Rabs (E–H) and total Rabs (I–L) levels. (M) GTP‐RAB10 and total RAB10 levels were assayed in brain homogenates from 16‐month‐old 2N, Dp16, and Dp16: *App+/+/‐ *mice. Quantitation and statistical analysis are shown in the lower panels (N, O). (P) The levels of CCZ1 and SH3BP5 were measured in the brain homogenates from 16‐month‐old 2N, Dp16, and Dp16: *App+/+/‐ *mice with β‐actin used as a loading control. Quantitation and statistical analysis are shown in the lower panels (Q, R). One‐way ANOVA followed by Newman–Keuls Multiple Comparison test; *n* = 4 for 2N, *n* = 3 for Dp16, and *n* = 3 for Dp16: *App+/+/‐ *in panels A–O; *n* = 3 for each group in panel P–R; *p < 0.05, **p < 0.01, ***p < 0.001. ANOVA, analysis of variance; APP, amyloid precursor protein; β‐CTF, the beta‐C‐terminal fragment; GEF, guanine‐nucleotide exchange factors; GTP, guanosine 5′‐triphosphate.

To directly determine whether increased *App* gene dose causes endosomal Rab dysregulation, we reduced the gene copy number in 16‐month‐old Dp16 mice from three to two (Dp16: *App+/+/‐  *mice). This restored fl‐APP and its CTFs, including β‐CTF, to 2N levels, without affecting the levels of the product of another triplicated gene, *Dyrk1a* (Figure 3A–D). Remarkably, RABs 5, 4, 7, 11, and 10 activity levels normalized in Dp16: *App+/+/‐ * mice (Figure 3A,E–L). Thus, the increased *APP* gene dose induces hyperactivation of RAB5 and downstream endosomal Rabs.

### 3.7 Endosomal Rab GEF/GAP imbalances induced by increased *App* gene dose in Dp16 mice

We compared GEFs and GEF/GAP ratios in Dp16 mice with those in 2N mice. In 4‐month‐old Dp16 mice forebrains, levels of the RAB5 GEF Rabex‐5 and its GAPs (USP6NL or SGSM3) were unchanged, as were levels of Rabaptin‐5, which enhances Rabex‐5 GEF activity^56^ (Figure S9A–D). Similar to DS brains, protein levels of RABs 7 and 11 GEFs CCZ1 and SH3BP5 were significantly increased, whereas their GAPs TBC1D2 and EVI5 remained unchanged. This led to higher CCZ1/TBC1D2 and SH3BP5/EVI5 GEF/GAP ratios (Figure S9E–L). No changes were observed in other RAB7 or RAB11 GAPs (TBC1D5, TBC1D11, and TBC1D15)^9^ or RAB3 GEF (MADD) and GAPs (Rab3GAP1 and Rab3GAP2) (Figure S9M–Q). Strikingly, the increases in CCZ1 and SH3BP5 were absent in the Dp16: *App+/+/‐* mouse (Figure 3P–R). Thus, increased *App* gene dose was necessary for increases in the activity of RABs 7 and 11 and the levels of their GEFs and GEF/GAP ratios.

### 3.8 Increased lysosomal cathepsin levels and activity induced by *App* gene dose

To determine if Dp16 mice replicate the changes in cathepsins observed in DS brains we measured cathepsin levels in the forebrains of both young (4 months) and aged (16 months) Dp16 mice. Dp16 mice showed increased levels of cathepsins B, L, and mature cathepsin D compared to 2N controls, with more pronounced increases at 16 months of age (Figure S10A–E). These changes were absent or reduced in 16‐month‐old Dp16: *App+/+/‐* mice (Figure S10D,E). To explore the underlying cause for these changes, we examined mRNA levels for these cathepsins and found them unchanged in 4‐month‐old 2N and Dp16 mice (Figure S10F). The protein levels of transcription factors (TFE3, TFEB, pS211‐TFEB) were also unchanged, and there were no differences in protein stability (Figure S10G–K).

To further examine the changes in cathepsins, we examined their cellular distribution. Cathepsins B, L, and D are primarily present in the lysosomes of healthy cells,^57^ but their distribution changes in several AD models.^47^, ^48^, ^58^ We isolated lysosomes from cortical neuron cultures using dextran‐coated magnetite beads and detected LAMP1, LIMP2, and RAB7 in the lysosomal fractions (lyso‐frs) of both Dp16 neurons and 2N controls, with minimal presence of markers for other organelles, such as RAB5 and EEA1 (early endosomes), KDEL (ER), and GM130 (cis‐Golgi). Cathepsins B, L, and D were localized in lysosomes in both Dp16 and 2N neurons, with increased levels evident in Dp16 (Figure S11A–D). Moreover, we measured cathepsin activities in the brain lysates. Significant increases in cathepsin B and L activity were observed in 9‐ to 10‐month‐old Dp16 mice, but no increases were seen in Dp16: *App+/+/‐* mice of the same age (Figure S11E). Using the DQ‐Red BSA (DQ‐BSA) probe to assess lysosomal degradative activity in cultured neurons, we found no differences in DQ‐BSA puncta intensity, but quantitative analysis showed a significant (≈30%) decrease in DQ‐BSA in Dp16 neurons. This decrease in available substrate precluded a firm conclusion regarding a possible correlation between cathepsin levels and lysosomal degradation (Figure S11F,G). The findings are evidence for increases in lysosomal cathepsin levels and activity in Dp16 mice and point to a necessity for increased *APP* gene dose.

### 3.9 Impact of RAB5 hyperactivation on downstream endosomal Rab activity and GEFs

Our findings and those of others^16^, ^17^, ^18^ suggest that increased β‐CTF leads to increases in RAB5 retention and activation on EEs. This β‐CTF‐dependent RAB5 hyperactivation likely enhances the activation of downstream endosomal Rabs. β‐CTF interacts with APPL1,^16^ thus serving to stabilize active RAB5 on EEs.^59^, ^60^ Mechanisms linking RAB5 to the activation of downstream Rabs^42^, ^61^ include the possibility that increased activated RAB5 binding to the membrane augments the recruitment of GEFs for RABs 7 and 11, resulting in their increased activation. To further explore this possibility, we examined the events upstream and downstream of RAB5 hyperactivation.

**FIGURE 4:**
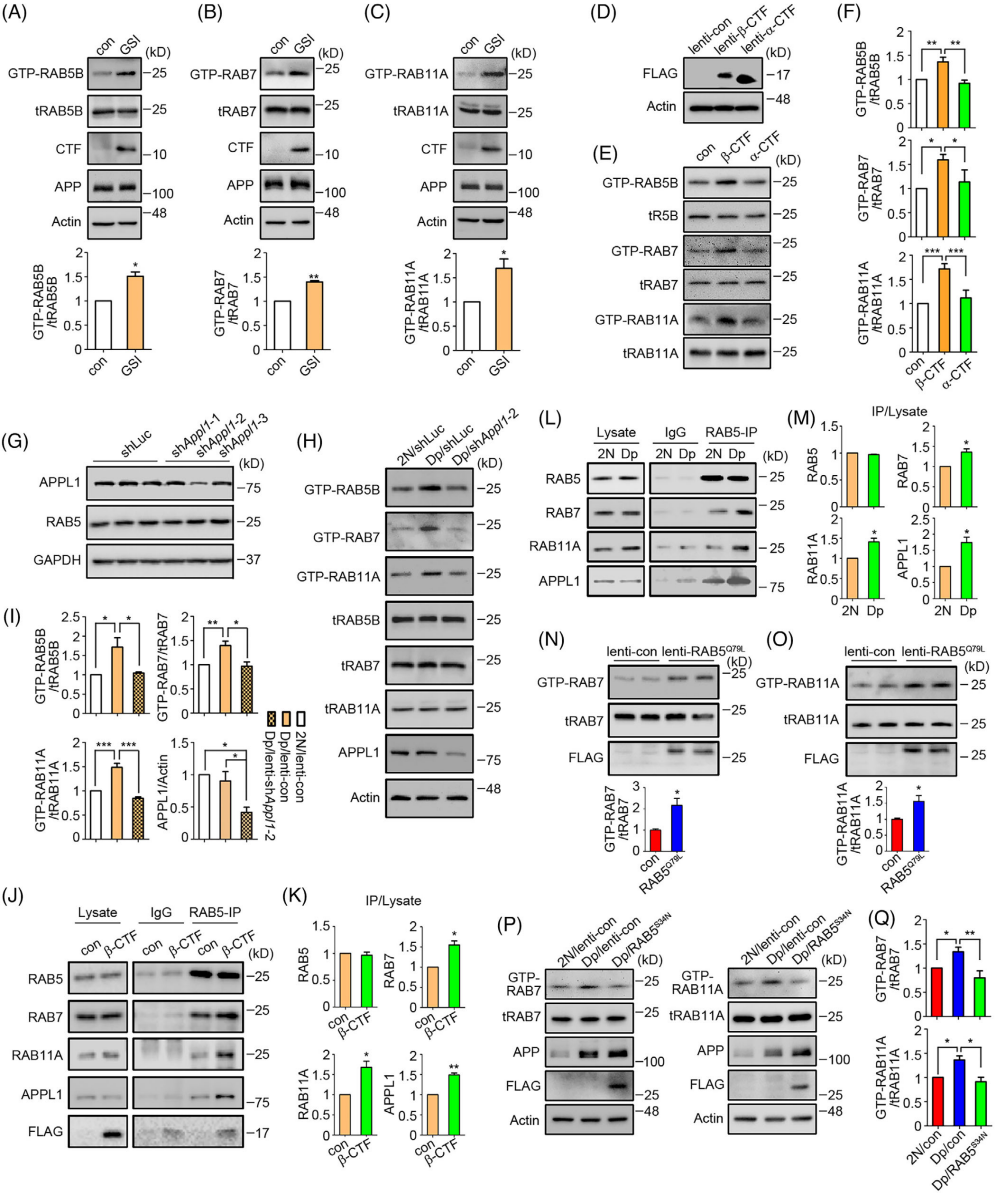
RAB5 hyperactivation is necessary for the widespread hyperactivation of endosomal Rabs in Dp16 neurons. (A–C) Primary wild‐type cortical neurons were treated with 1 µM GSI for 24 h followed by GTP agarose pull‐down assay to analyze the activities of RABs 5, 7, and 11. *N* = 3 for RAB5B and RAB7; *n* = 5 for RAB11A. (D) Evaluation of lentivirus‐mediated β‐CTF or α‐CTF expression in wild‐type neurons. (E, F) Primary cortical neurons were infected with β‐CTF or α‐CTF lentivirus for 72 h, followed by GTP agarose pull‐down assay to analyze the activities of RABs 5, 7, and 11. *N* = 4 for RAB5B and RAB7; *n* = 5 for RAB11A. (G) Screening of sh*Appl1* by infecting primary cortical neurons with lentivirus expressing shRNAs targeting mouse *Appl1* for 72 h. (H, I) Primary 2N or Dp16 cortical neurons at DIV5 were infected with sh*Appl1*‐2 or control lentivirus for 72 h to assess RABs 7 and 11 activities. APPL1 was also probed. *N* = 3 for RAB5B, RAB7, and APPL1; *n* = 4 for RAB11A. (J) Primary neurons were infected with either β‐CTF lentivirus or control lentivirus. Subsequently, endosomes were immunoprecipitated using a RAB5‐specific antibody or IgG. The levels of RABs and APPL1 in the immunoprecipitants were evaluated (K). *N* = 3. (L, M) Endosomes were immunoprecipitated with the same RAB5 antibody from either 4‐month‐old 2N or Dp16 brains followed by evaluation of RABs and APPL1 in the immunoprecipitants. *N* = 3. (N, O) Primary wild‐type cortical neurons were infected with RAB5^Q79L^ lentivirus for 48 h, followed by GTP agarose pull‐down assay to assess RABs 7 and 11 activities. *N* = 5 in lenti‐con, *n* = 6 in lenti‐RAB5^Q79L^ for RAB7; *n* = 6 in both lenti‐con and lenti‐RAB5^Q79L^ for RAB11A. (P) Primary cortical neurons from 2N or Dp16 embryos at DIV5 were infected with RAB5^S34N^ or control lentivirus for 72 h to assess RABs 7 and 11 activities. APP was also probed. Quantitation and statistical analysis are shown in the lower panel Q. *N* = 4 in each group for RAB7; *n* = 3 in each group for RAB11A. Paired *t*‐test for A–C, K, M; unpaired *t*‐test for N, O; one‐way ANOVA followed by Newman–Keuls Multiple Comparison test for F, I, Q; *p < 0.05, **p < 0.01, ***p < 0.001. ANOVA, analysis of variance; APP, amyloid precursor protein; β‐CTF, the beta‐C‐terminal fragment; GSI, γ‐secretase inhibitor; GTP, guanosine 5′‐triphosphate; shRNA, short hairpin RNA; DIV, days in vitro.

**FIGURE 5:**
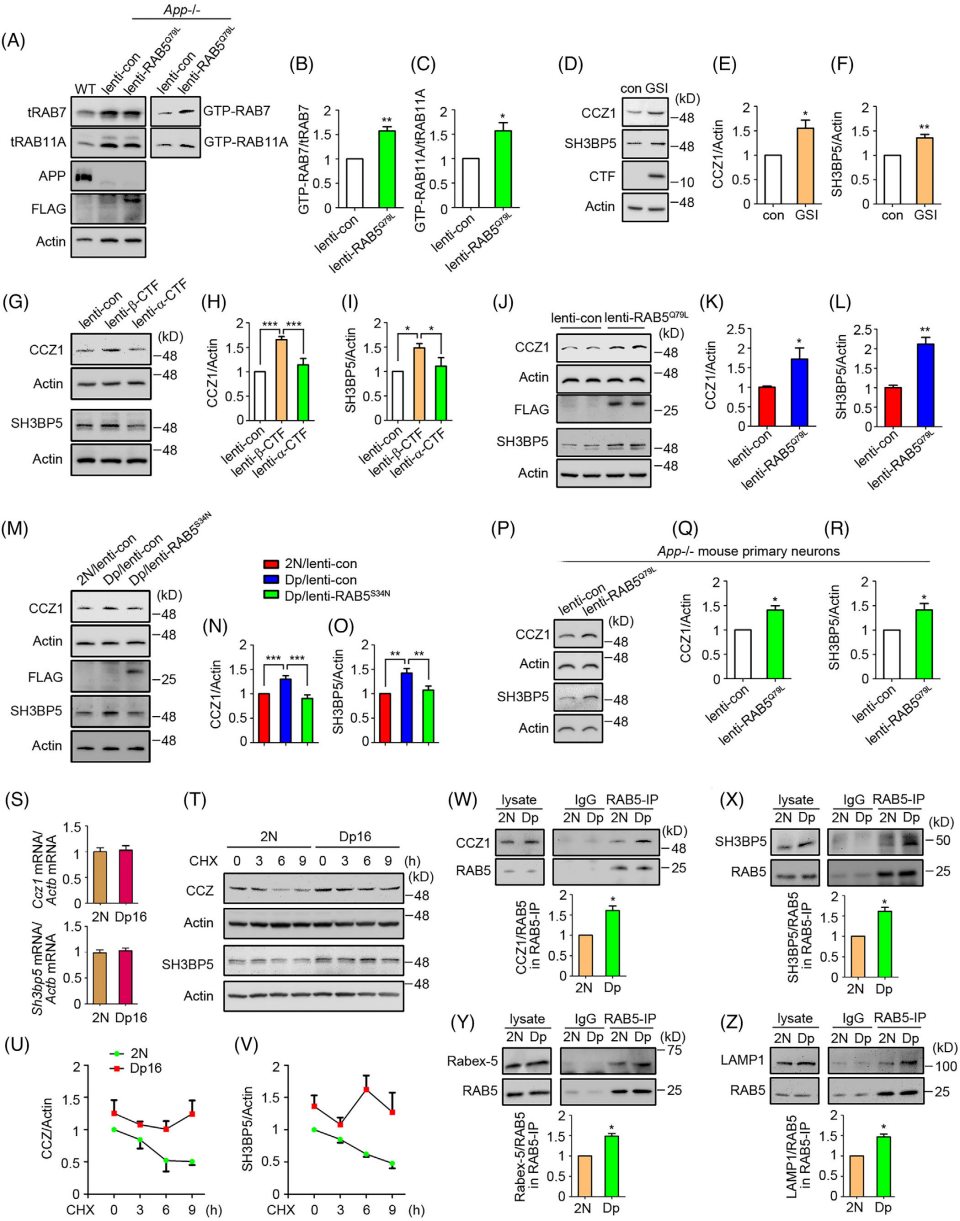
RAB5 hyperactivation is necessary for upregulating GEFs of RABs 7 and 11 in Dp16 neurons. (A) Primary *App−*/− cortical neurons were infected with RAB5^Q79L^ lentivirus for 48 h, followed by GTP agarose pull‐down assay to assess RABs 7 and 11 activities. Wild‐type neurons were lysed to assess the expression of APP. (B, C) Quantitation and statistical analysis of RABs 7 and 11 activities in A. *N* = 5 for each group. (D) The expression levels of CCZ1 and SH3BP5 in GSI‐treated (1 µM GSI, 24 h) wild‐type neurons were measured. (E, F) Quantitation and statistical analysis of the levels of CCZ1 and SH3BP5 in D. *N* = 5 in each group. (G) The expression levels of CCZ1 and SH3BP5 in the β‐CTF or α‐CTF lentivirus‐infected wild‐type neurons were measured. (H, I) Quantitation and statistical analysis of the levels of CCZ1 and SH3BP5 in G. *N* = 7 in each group for CCZ1; *n* = 4 for SH3BP5. (J) The expression levels of CCZ1 and SH3BP5 in RAB5^Q79L^ lentivirus‐infected neurons were measured. (K, L) Quantitation and statistical analysis of the levels of CCZ1 and SH3BP5 in J. *N* = 7 in each group for CCZ1; *n* = 4 for SH3BP5. (M) The expression levels of CCZ1 and SH3BP5 in the RAB5^S34N^ or control lentivirus‐infected 2N or Dp16 cortical neurons were measured. (N, O) Quantitation and statistical analysis of the levels of CCZ1 and SH3BP5 in M. *N* = 8 in each group for CCZ1; *n* = 6 for SH3BP5. (P) *App*−/− cortical neurons were infected with RAB5^Q79L^ lentivirus for 48 h to assess the levels of CCZ1 and SH3BP5. *N* = 5 for each group. (Q, R) Quantitation and statistical analysis of the levels of CCZ1 and SH3BP5 in P. (S) The mRNA levels of *Ccz1* and *Sh3bp5* were analyzed in the brains of 4‐month‐old 2N and Dp16 mice using qPCR. *N* = 5 in 2N and Dp16 for *Ccz1*; *n* = 4 in 2N, *n* = 5 in Dp16 for *Sh3bp5*. (T) Assessment of the decay rate of CCZ1 and SH3BP5 in 2N and Dp16 cortical neurons in the presence of 100 µg/mL CHX for the indicated durations. (U, V) Quantitation and statistical analysis of the decay rates of CCZ1 and SH3BP5 in T. *N* = 3 in each group for CCZ1; *n* = 4 in each group for SH3BP5. (W–Z) Endosomes were immunoprecipitated with a RAB5 antibody from either 4‐month‐old 2N or Dp16 brains followed by evaluation of CCZ1, SH3BP5, Rabex‐5, and LAMP1 in the immunoprecipitants. *N* = 3 in each group. Paired Student *t*‐test for B, C, E, F, Q, and R; unpaired Student *t*‐test for K and L; one‐way ANOVA followed by Newman–Keuls Multiple Comparison test for H, I, N, and O; *p < 0.05, **p < 0.01, ***p < 0.001. ANOVA, analysis of variance; APP, amyloid precursor protein; β‐CTF, the beta‐C‐terminal fragment; CHX, cycloheximide; GEF, guanine‐nucleotide exchange factors; GTP, guanosine 5′‐triphosphate; qPCR, quantitative polymerase chain reaction.

WT cortical neurons treated with γ‐secretase inhibitor (GSI) compound E showed elevated CTF levels and increased activation of RABs 5, 7, and 11 (Figure 4A–C). Expression of β‐CTF but not α‐CTF in neurons also increased these Rab activities (Figure 4D–F). Reducing APPL1 levels in Dp16 neurons by shRNA prevented hyperactivation of endosomal Rabs (Figure 4G–I). It is important to note that increased levels of RABs 7 and 11 were detected in RAB5 immunoprecipitates, which were enriched RAB5 using a RAB5‐specific antibody from WT neurons that overexpressed β‐CTF (Figure 4J,K). We obtained the same result comparing 4‐month‐old Dp16 to 2N brains (Figure 4L,M). Finally, increased APPL1 on RAB5‐containing membranes was observed in both β‐CTF‐overexpressing neurons and Dp16 mice (Figure 4J–M). Reinforcing these observations, Dp16 neurons consistently exhibited increased co‐localization of transfected RAB5 with RABs 4, 7, and 11 compared to 2N controls (Figure S12). Attesting to the essential role of RAB5 hyperactivation on downstream events, constitutively active RAB5 (RAB5^Q79L^) expression in WT neurons increased RABs 7 and 11 activation, whereas a dominant‐negative RAB5 (RAB5^S34N^) normalized RABs 7 and 11 activity in Dp16 neurons (Figure 4N–Q). This effect was independent of APP, as shown in *App*−/− neurons (Figure 5A–C). Thus we conclude that increased β‐CTF levels act through APPL1 to increase RAB5 activation, which is sufficient to induce increased endosomal recruitment and activation of RABs 7 and 11.

Next, we asked what events link RAB5 hyperactivation to endosomal GEFs for RABs 7 and 11. GSI treatment (Figure 5D–F) and β‐CTF overexpression (Figure 5G–I) increased RABs 7 and 11 GEF levels in WT neurons. Overexpression of RAB5^Q79L^ in WT neurons also increased GEF levels (Figure 5J–L), whereas RAB5^S34N^ expression in Dp16 neurons normalized RAB7 and RAB11 GEFs (Figure 5M–O). Moreover, RAB5^Q79L^ also increased CCZ1 and SH3BP5 in *App−/−* neurons (Figure 5P–R) demonstrating that APP expression was not required for this change. Thus RAB5 activity is directly linked to the levels of the GEFs for RABs 7 and 11.

How increased RAB5 activity increases RAB7 and RAB11 GEF levels was next examined. mRNA levels remained unchanged in the Dp16 brain (Figure 5S), but both CCZ1 and SH3BP5 were more stable in Dp16 neurons, showing minimal reduction over 9 h with CHX (Figure 5T–V). GEF binding to endosomal membranes mediates downstream RAB activation.^42^ We reasoned that increased membrane binding could enhance protein stability. Support for this idea was a 50% increase in CCZ1 and SH3BP5 binding to RAB5 membranes in 4‐month‐old Dp16 brains; it was noteworthy that Rabex‐5 was also increased (Figure 5W–Y). Of interest, increased LAMP1 in RAB5 immunoprecipitated from Dp16 brains suggests an enhanced fusion of EEs with lysosomal compartments (Figure 5Z), similar to observations in studies using RAB5^Q79L^ transfection.^62^ Our findings are consistent with a model in which RAB5‐mediated increases in membrane binding of the GEFs for RABs 7 and 11, leading to their increased activation.

### 3.10 Impact of RAB5 hyperactivation on the levels and activity of lysosomal cathepsins

**FIGURE 6:**
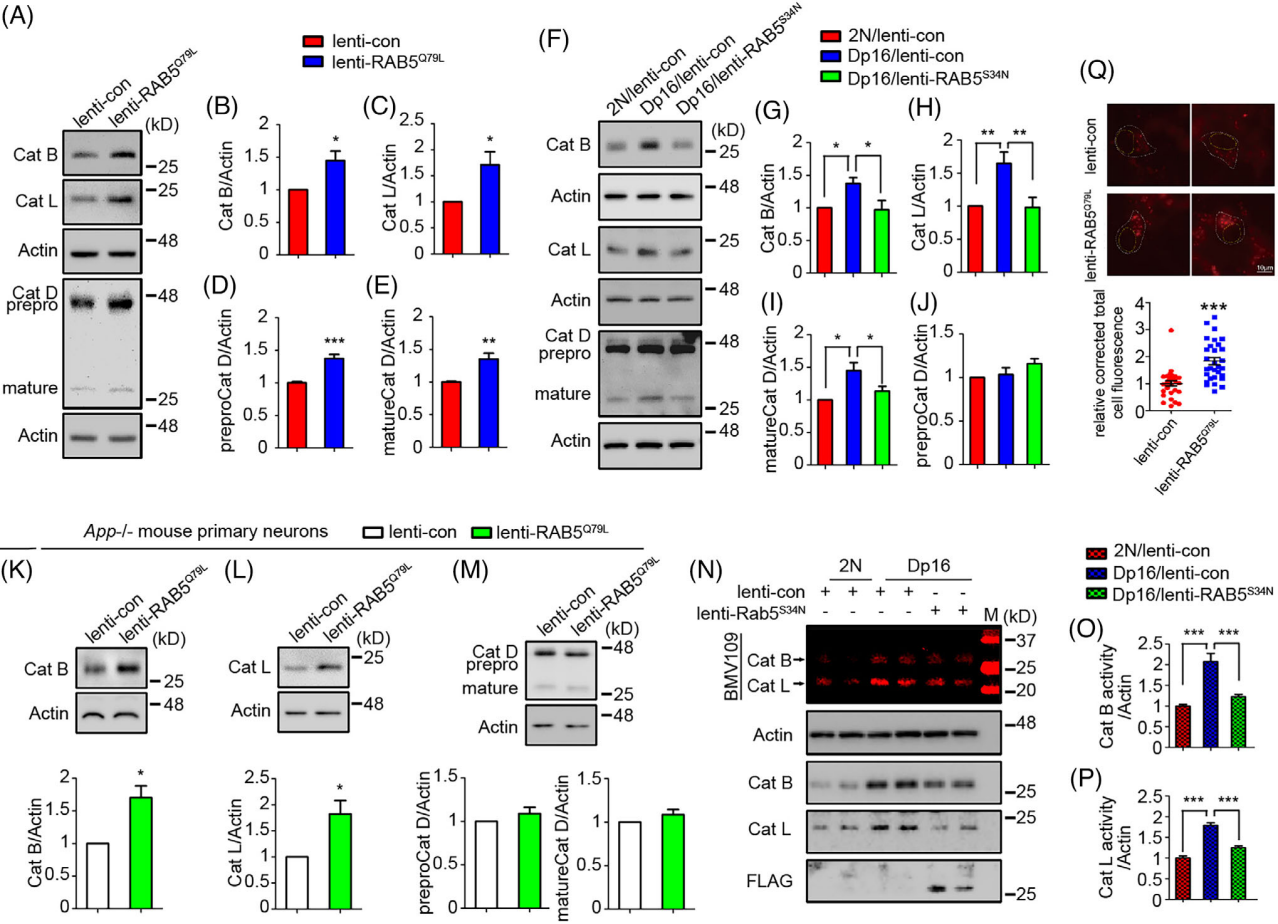
RAB5‐dependent changes in the levels and activities of cathepsins in Dp16 neurons. (A) The levels of cathepsins in wild‐type cortical neurons expressing RAB5^Q79L^ for 48 h were measured. (B–E) Quantitation and statistical analysis of the cathepsin levels in A. *N* = 5 in lenti‐con and lenti‐RAB^Q79L^ for Cat B and Cat L, *n* = 8 for Cat D. (F) The levels of cathepsins in 2N and Dp16 cortical neurons expressing RAB5^S34N^ or control lentivirus for 72 h were measured. (G–J) Quantitation and statistical analysis of the cathepsin levels in F. *N* = 5 in each group for Cat B, *n* = 6 for Cat L, n = 4 for Cat D. (K–M) The levels of cathepsins in *App−*/− cortical neurons expressing RAB5^Q79L^ for 48 h were measured. *N* = 5 in lenti‐con and lenti‐RAB^Q79L^ for Cat B and Cat L, *n* = 4 for Cat D. (N) The activities of Cat B and Cat L in 2N, Dp16, and Dp16 expressing RAB5^S34N^ were assessed by fluorescent SDS‐PAGE cysteine cathepsin activity profiling using BMV109. (O, P) Quantitation and statistical analysis of the cathepsin activities in N. *N* = 6 in each group for Cat B and Cat L. (Q) Live imaging of DQ‐BSA in the lenti‐RAB5^Q79L^ or lenti‐control (48 h) infected wild‐type cortical neurons. *N* = 30 cells in lenti‐con and lenti‐RAB^Q79L^ from three independent experiments. Unpaired Student *t*‐test for B–E, K–M, Q; one‐way ANOVA followed by Newman–Keuls Multiple Comparison test for G–J, O, and P; *p < 0.05, **p < 0.01, ***p < 0.001. ANOVA, analysis of variance; Cat, cathepsin; SDS‐PAGE, sodium dodecyl sulfate–polyacrylamide gel electrophoresis.

We asked if RAB5 hyperactivation alone could increase cathepsin levels. Expression of RAB5^Q79L^ in WT neurons significantly elevated levels of cathepsins B, L, and D (Figure 6A–E). Conversely, RAB5^S34N^ expression in Dp16 neurons normalized cathepsin levels (Figure 6F–J). In *App−/−* neurons, RAB5^Q79L^ increased cathepsins B and L, but not D (Figure 6K–M). Assessing cysteine cathepsin activity profiling using BMV109 in fluorescent SDS‐PAGE^32^ revealed increased activities of cathepsins B and L in Dp16 neurons, which were normalized by RAB5^S34N^ expression (Figure 6N–P). In addition, RAB5^Q79L^ expression in WT neurons significantly intensified the DQ‐BSA‐positive puncta, indicating enhanced lysosomal degradation (Figure 6Q). Thus, although increased *App* gene expression was necessary, RAB5 hyperactivation was both necessary and sufficient to increase cathepsin levels in Dp16 neurons.

## 4 DISCUSSION

The ELN maintains neuronal homeostasis by degrading proteins, lipids, and organelles while supporting signaling and synaptic function.^63^, ^64^ ELN dysregulation is featured prominently in various neurodegenerative disorders,^65^ including AD and DS‐AD. Our study in the DS brain demonstrates widespread ELN dysregulation, showing increased activities of endosomal RABs 4, 5, 7, and 11 and elevated levels and activities of cathepsins B and L; results from the Dp16 model largely recapitulated those identified in DS brains. In both DS and Dp16 brains, the changes were a function of increased *APP* gene dose (Table S8). Both in vitro and in vivo studies supported a mechanism wherein RAB5 hyperactivation served as the central force for disrupting the ELN and compromising neuronal function, and suggested a novel mechanism under which RAB5 hyperactivation disrupts the endosomal Rab cascade by increasing the levels and membrane binding of the GEFs for RABs 7 and 11.

RAB5 plays a defining regulatory role in the ELN,^66^ with evidence supporting increased RAB5 activity and ELN dysfunction in AD and DS.^16^, ^17^, ^23^ Enlarged EEs have been reported in AD, FAD, and DS‐AD.^15^, ^16^, ^17^, ^18^, ^23^, ^33^, ^38^ EE enlargement correlated with larger RAB7‐positive LEs in DS fibroblast,^33^ along with upregulated lysosomal biogenesis,^11^ defective lysosomal acidification, reduced protein turnover, and increased lysosomal enzyme levels.^11^, ^67^, ^68^ Model systems have further shown that RAB5 hyperactivation disrupted the axonal transport of trophic signals.^17^, ^23^

Neurodegeneration models have been used to decipher and target pathogenic mechanisms. Effective models must demonstrate: (1) construct validity, which speaks to common physiological or biological basis(es); (2) face validity, which addresses the presence of biologically and clinically meaningful measures; and (3) predictive validity, which focuses on whether a specific intervention will have a comparable impact in the model and humans. The Dp16 model has shown construct and face validity through *APP* gene dose effects on synaptic proteins, retromer protein levels, neuron vulnerability, tau pathology, and inflammation.^39^, ^69^, ^70^ Brain tissue experiments in Dp16 mice mirrored ELN phenotypes of DS and DS‐AD, affirming the model's utility for pathogenesis and treatment studies. Future studies will address whether the Dp16 model can also be used to support tests of predictive validity through treatments directed against APP and/or RAB5 to ameliorate ELN disruption in DS and DS‐AD.

Although previous studies have revealed that β‐CTF elevation led to RAB5 hyperactivation and DS‐AD‐relevant degenerative phenotypes,^16^, ^17^, ^40^, ^49^ it was not clear whether physiologically relevant increases in RAB5 activity, as detected in DS brains, were directly responsible and how RAB5 hyperactivity led to ELN dysregulation. Our study provides critical evidence for a pathway wherein RAB5 hyperactivation triggers increased activation of downstream endosomal Rabs. Our findings indicate that RAB5 activity results in (1) recruitment of RABs 7 and 11 GEFs to RAB5‐containing membranes; (2) continued presence thereon of increased Rabex‐5, the GEF for RAB5; (3) GEF stabilization, with increased binding to RAB5‐positive endosomes; (4) increased RABs 7 and 11 binding to these membranes; and (5) increased levels of the lysosomal protein LAMP1. Under normal conditions, RAB5 regulates ELN function via a cascade involving GEF recruitment and activation of downstream endosomal Rabs. In the case of RAB5–RAB7 conversion, RAB5 mediates membrane recruitment of the RAB7 GEF CCZ1‐MON1, which then acts to effect EE maturation to LEs by recruiting and activating RAB7, followed by the coordinated release by activated MON1 of Rabex‐5 and RAB5.^42^, ^71^ Our results demonstrate disruption of the endosomal Rab cascade through increased levels of RABs 7 and 11 binding to RAB5‐positive endosomal membranes. Results from previous studies have documented a critical role for RAB5 in showing that reducing its level below a threshold collapses the ELN, thereby diminishing EEs, LEs, and lysosomes.^66^ Evidence from our study and others show that increased activation of RAB5 may not simply expand the ELN but instead acts to induce failed maturation of endosomal compartments downstream from the early endosome. Indeed, studies with RAB5^Q79L^ have also showed increased co‐localization with LE and lysosome markers, indicating disruption of the pathway.^62^ We conclude that increased RAB5 activity can lead to disruption of the normal Rab cascade. Our Dp16 model and DS brain findings are consistent with this formulation. They show that even a modest increase in RAB5 activity disrupts the RAB5‐initiated cascade. We suggest that β‐CTF‐induced persistent membrane occupancy inhibits RAB5 release, analogous to RAB5^Q79L^ effects.^72^ It is important to note that the increases in RAB5 activity were not accompanied by increases in total Rabex‐5 or Rabaptin‐5, arguing against changes in the levels of these regulatory proteins. It will be important to explore the underlying mechanisms, including the failure of MON1 to release RAB5 and Rabex‐5. Further studies designed to elucidate the impact of RAB5 hyperactivation on endosomal trafficking, signaling, degradation, and neurodegeneration will also be needed. Indeed, it is imperative to explore how failed EE maturation and ELN dysregulation contribute to DS‐AD pathogenesis.

Post‐mortem human brain studies have several limitations. These tissues cannot capture dynamic, time‐dependent biological processes, such as disease progression. Tissue quality may be influenced by factors such as variable post‐mortem intervals, storage conditions, and the presence of secondary comorbidities. In addition, confounding variables, including age, medication history, and lifestyle factors, can complicate data interpretation. However, the relatively large sample size and narrow age range in our study strengthen the robustness of our findings. The minimal protein degradation across the samples further reinforces the validity of the results. Finally, ethical and logistical challenges in obtaining sufficient high‐quality samples, particularly for rare cases such as PT, may limit the generalizability of the results. It has been noted that apolipoprotein E (*APOE*) ε4 accelerates endosome abnormalities in early but not moderate to severe sporadic AD.^38^ Unfortunately, we are unable to evaluate the impact of *APOE* ε4 in our DS‐AD and DS samples due to the lack of detailed genetic information, although our samples are from patients at an advanced stage. Further research is needed on whether *APOE* genotype affects ELN in DS.

Our study demonstrates that the Dp16 mouse model mirrors ELN changes observed in DS brains. The underlying mechanism involves increased *APP* gene dose, leading to increased levels of APP β‐CTF with RAB5 hyperactivation. The findings in post‐mortem human brain, the Dp16 mouse model brain, and in vitro mechanistic studies combine to support our hypothesis that in DS increased *APP* gene dose–induced RAB5 hyperactivation acts as the central hub for dysregulation of endolysosomal function, changes which appear to contribute to AD‐linked pathogenesis in DS. Targeting *APP* or *Rab5* gene expression may be a feasible approach to reverse endolysosomal phenotypes associated with DS‐AD and perhaps in related conditions.

## CONFLICT OF INTEREST STATEMENT

W.C.M. serves as a Scientific Advisory Board (SAB) member and holds stock options from Alzheon, Inc. and Promis, Inc. W.C.M. also serves as an SAB member and holds stock in Acta Pharmaceuticals, Inc. His name is on a patent under University of California San Diego and Massachusetts General Hospital concerning γ‐secretase modulators licensed to Acta Pharmaceuticals, Inc. He has served as a consultant to AC Immune. W.C.M. holds a leadership position in the Trisomy 21 Research Society. He serves on committees for the Alzheimer's Project San Diego and the American Neurological Association and an NIH COBRE Grant to the University of Nebraska. W.C.M. received a royalty payment under a patent held by Stanford University licensed to Curasen. Author disclosures are available in the Supporting Information.

## CONSENT STATEMENT

All the post‐mortem tissues were donated by individuals or families who have given prior consent for scientific research.

## Supporting information



Supporting Information

Supporting Information

## Data Availability

Raw immunoblot images related to the data presented in this study are available upon request.
