# Supplementary material for: Hyperactivation of RAB5 disrupts the endosomal Rab cascade leading to endolysosomal dysregulation in Down syndrome: A necessary role for increased APP gene dose
Source: Alzheimers Dement. 2025 May 7;21(5):e70046. doi: 10.1002/alz.70046 (PMC12058456; doi:10.1002/alz.70046)
Supplement: Supplementary file 1 — Supporting Information [file ALZ-21-e70046-s001.docx]

**Supplementary Figures**


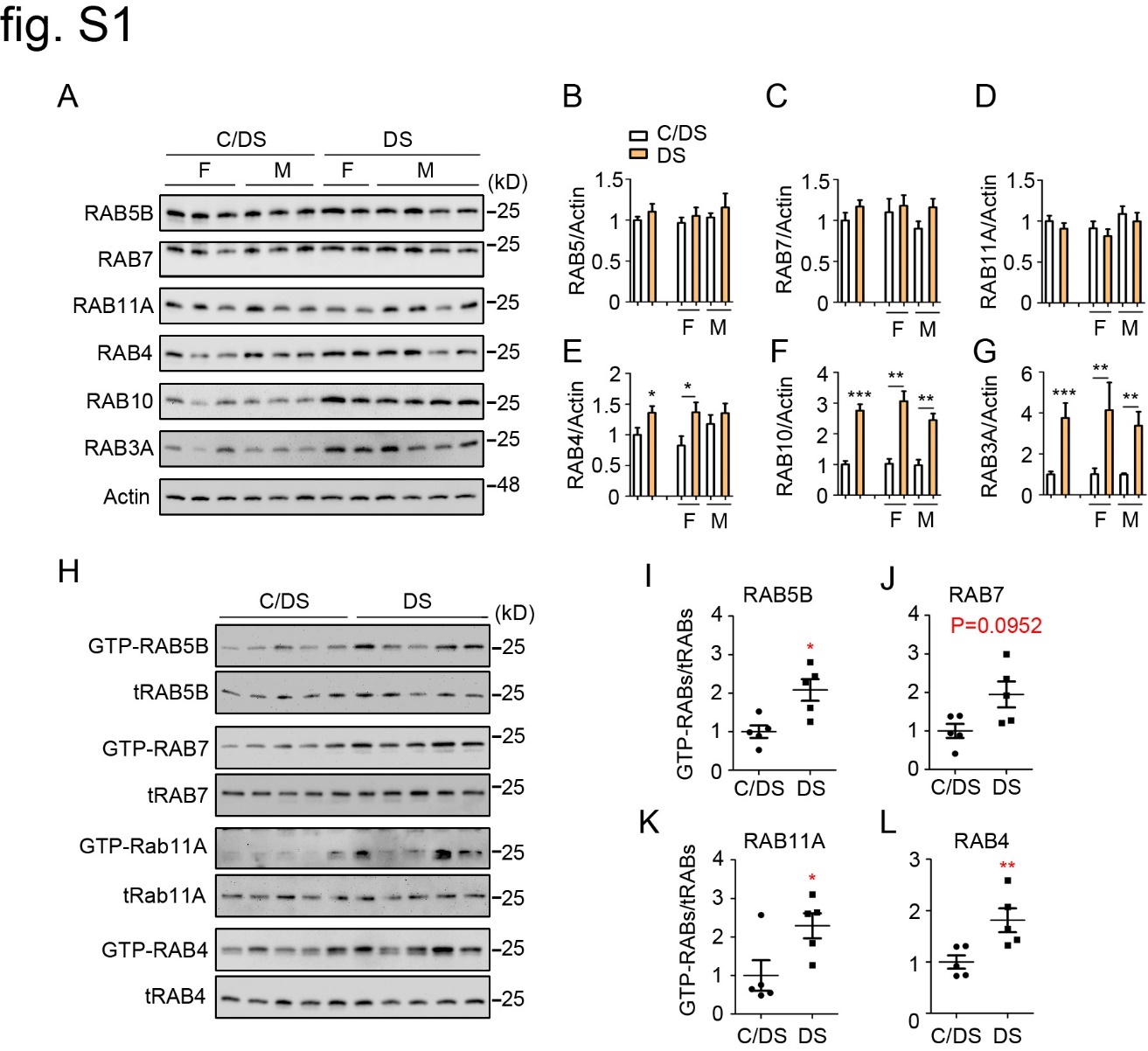


**Figure S1. Widespread hyperactivation of endosomal Rabs in non-demented DS frontal cortex.** (A) Western blotting of the levels of different Rabs in protein extracts from the frontal cortex of patients with DS and C/DS. β-Actin was used as a loading control. (B-G) Quantitation and statistical analysis of the levels of various Rabs in combined, females and males from DS and C/DS samples. (H) The activities of RABs 5, 4, 7, and 11 in the frontal cortex of DS and C/DS were measured by GTP agarose pull-down assay. (I-L) Quantitation and statistical analysis of the activities of different Rabs in DS and C/DS. Mann-Whitney test; F, female; M, male; n = 12 for C/DS (F: 6, M: 6), n = 12 for DS (F: 6, M: 6) for A to G; n = 5 for both C/DS and DS for H-L; **P* < 0.05, ***P* < 0.01, and ****P* < 0.001.


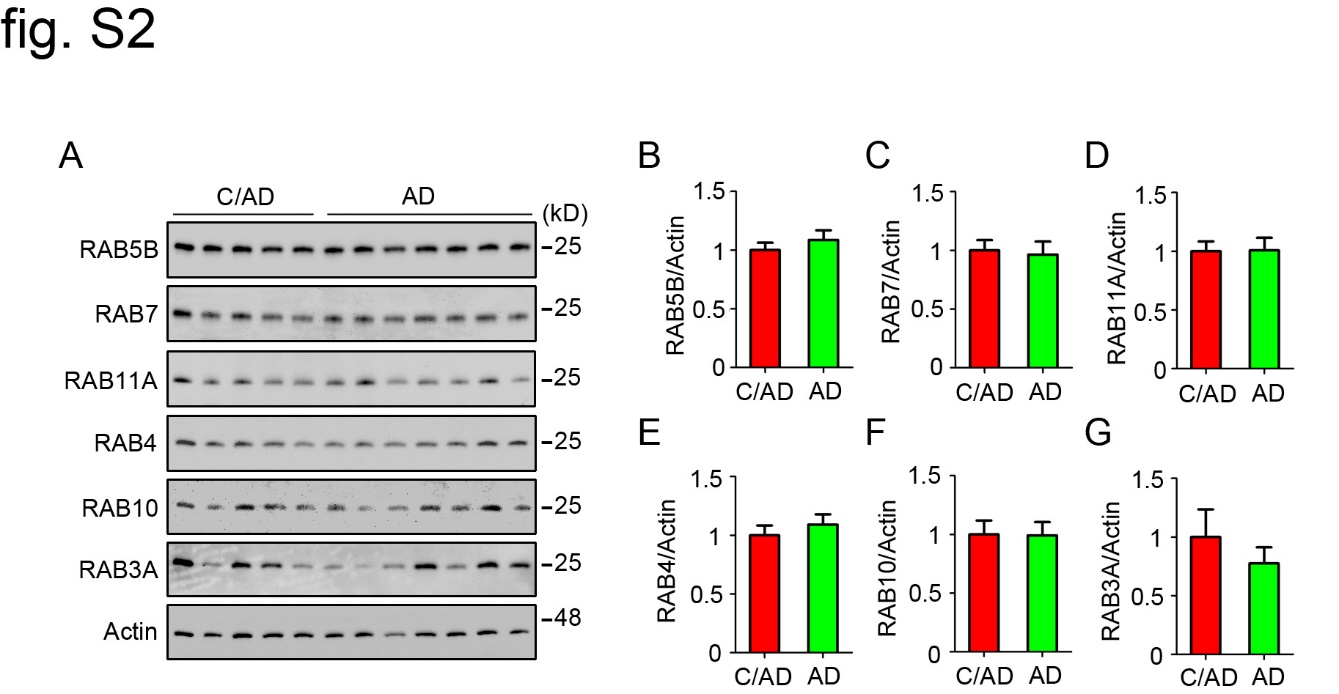


**Figure S2. The levels of endosomal Rabs in the frontal cortex of AD subjects and controls.** (A) Western blotting of the levels of different Rabs in protein extracts from the frontal cortex of patients with AD and C/AD. β-Actin was used as a loading control. (B-G) Quantitation and statistical analysis of the levels of various Rabs in AD and C/AD samples. n = 11 for C/AD; n = 14 for AD.


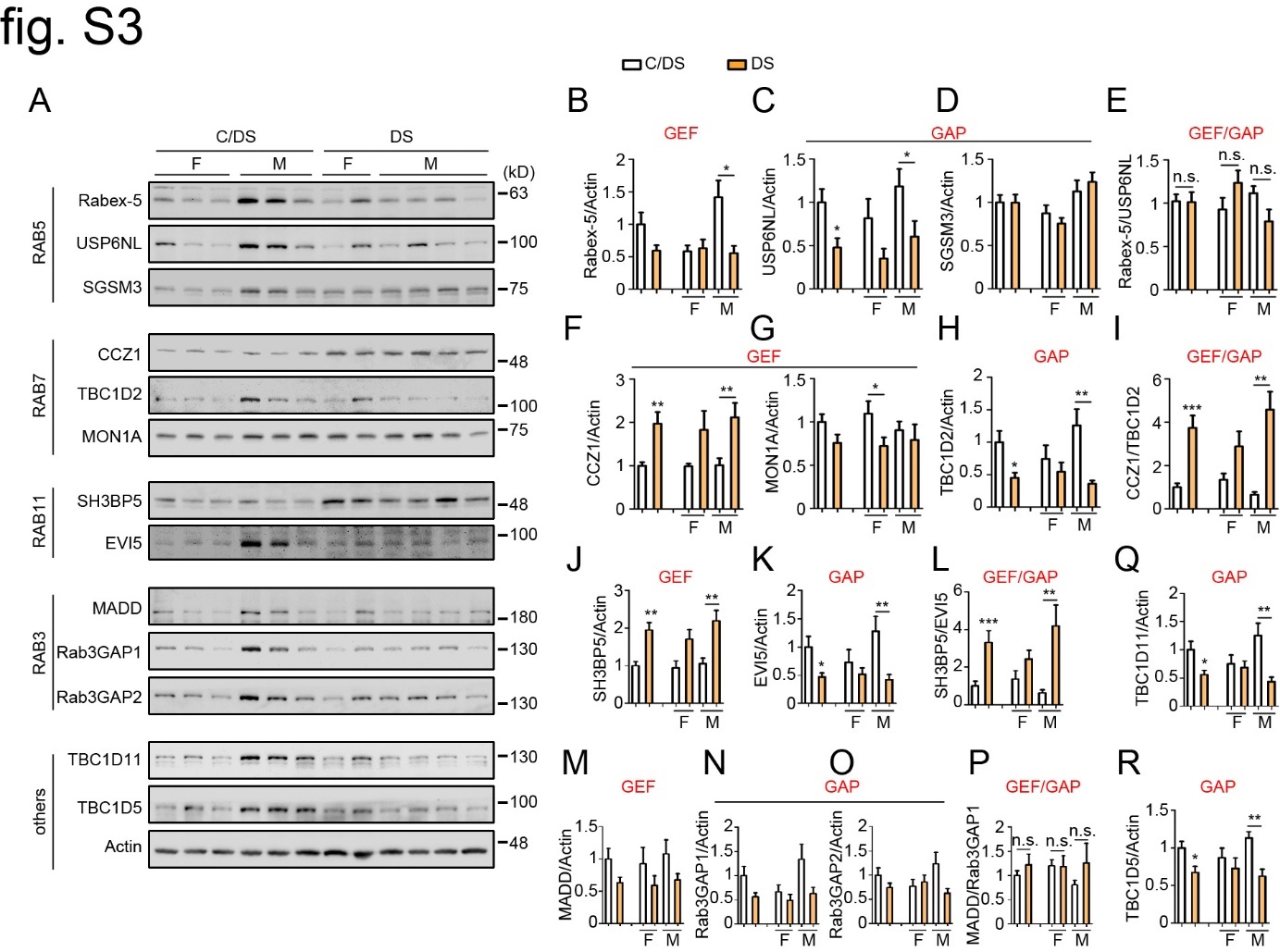


**Figure S3. Selective alterations of the GEFs of endosomal Rabs in non-demented DS frontal cortex.** (A- E) The levels of GEFs/GAPs of RAB5 (A, B-E), RAB7 (A, F-I), RAB11 (A, J-L), and RAB3 (A, M-P) were measured in the frontal cortex of DS and C/DS. Quantitation and statistical analysis were displayed on the right panels. (Q, R) The levels of other GAPs TBC1D11 and TBC1D5 were also analyzed in these samples. Mann-Whitney test; F, female; M, male; n = 12 for C/DS (F: 6, M: 6), n = 12 for DS (F: 6, M: 6); **P* < 0.05, ***P* < 0.01, ****P* < 0.001.


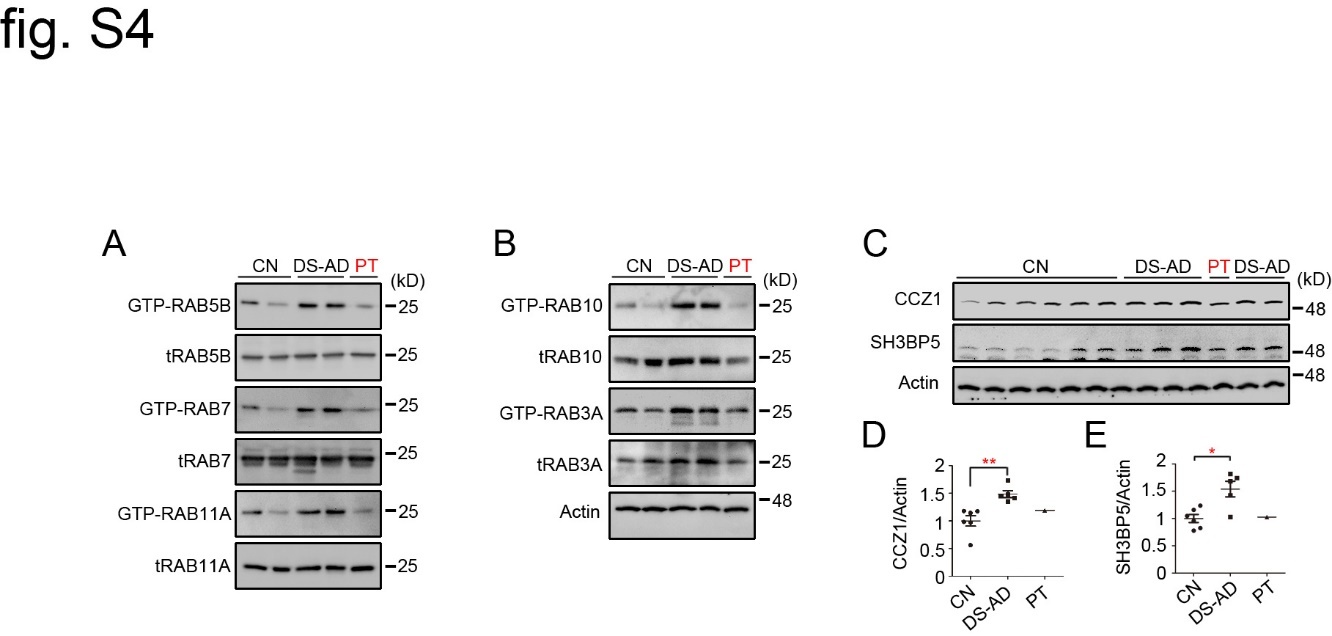


**Figure S4. Rab activity and GEF level assessment in the frontal cortex of the PT case.** (A, B) GTP agarose pull-down assay was used to measure the activities of RABs 5, 7, 11, 10, and 3A in the frontal cortex of the PT case compared to DS-AD and cognitive normal controls (CN). The levels of different Rabs in protein extracts were also measured. β-Actin was used as a loading control. (C) The levels of CCZ1 and SH3BP5 were measured in the frontal cortex of the PT case compared to DS-AD and CN. (D, E) Quantitation and statistical analysis of the levels of CCZ1 and SH3BP5 in C. Mann-Whitney test; **P* < 0.05, ***P* < 0.01.


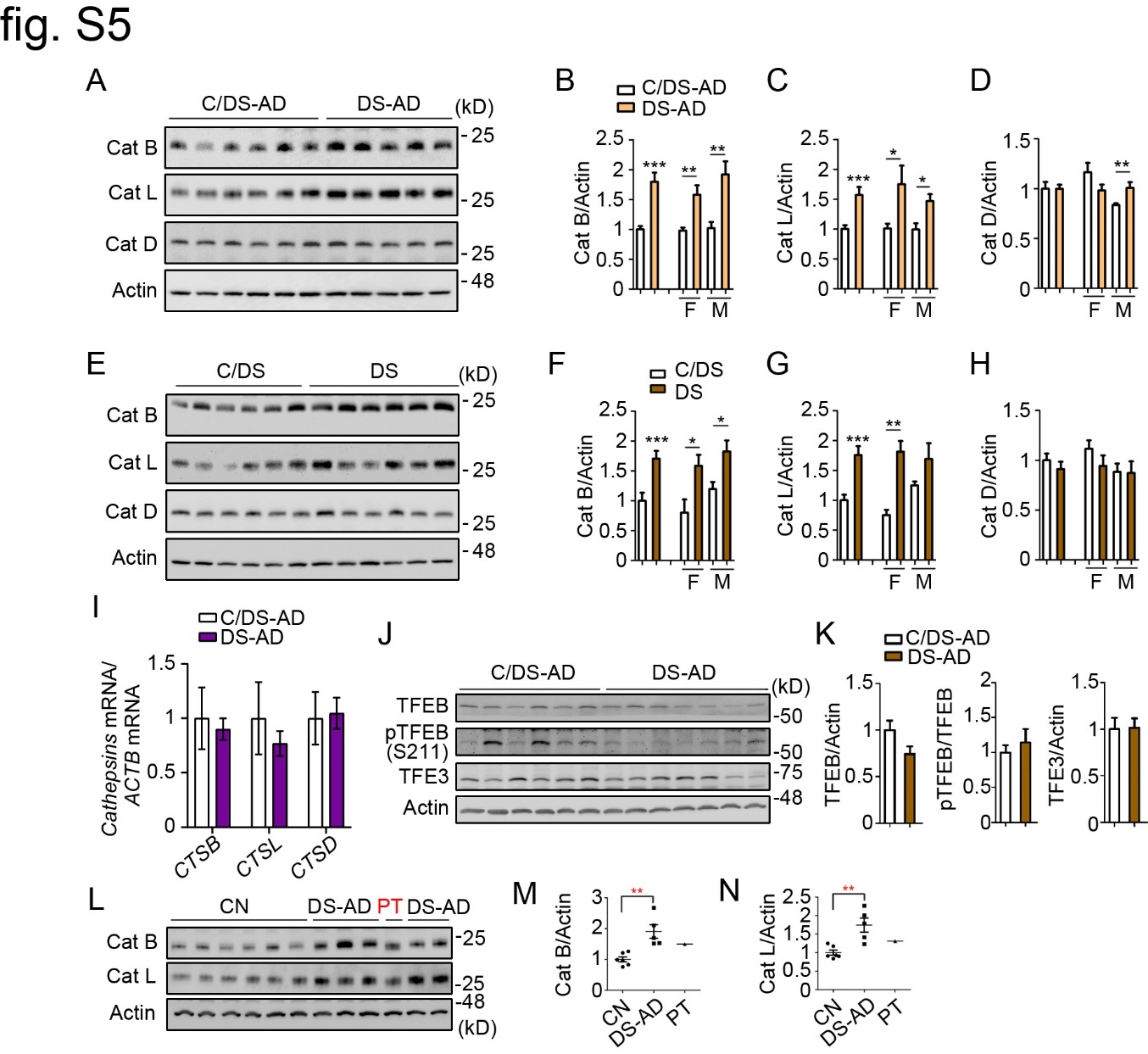


**Figure S5. The protein and mRNA levels of cathepsins in the frontal cortex of DS-AD and non-demented DS subjects as well as the PT-DS case.** (A, E) Western blot analysis of the levels of cathepsins B, L, and D in the frontal cortex of DS-AD and C/DS-AD and DS and C/DS with β-Actin as a loading control. Quantitation and statistical analysis of the levels of various Rabs in combined, females and males from DS-AD, C/DS-AD (B-D), DS, and C/DS samples (F-H). (I) The relative mRNA levels of *cathepsins* in the frontal cortex of DS-AD and C/DS-AD were assessed by qPCR. *ACTB* mRNA was used as an internal control. (J) Western blot analysis of the levels of transcription factors TFE3, TFEB, and pTFEB (S211) in the frontal cortex of DS-AD and C/DS-AD. Quantitation and statistical analysis were displayed in panel K. (L) The levels of cathepsins B and L were measured in the frontal cortex of the PT case compared to DS-AD and CN. (M, N) Quantitation and statistical analysis of the cathepsin levels in L. Mann-Whitney test; F, female; M, male; n = 12 for C/DS-AD (F: 6, M: 6), n = 11 for DS-AD (F: 4, M: 7) in A-D; n = 12 for C/DS (F: 6, M: 6), n = 12 for DS (F: 6, M: 6) in E-H; n = 6 for C/DS-AD, n = 6 for DS-AD in I; n = 12 for C/DS-AD, n = 13 for DS-AD in J-K; **P* < 0.05, ***P* < 0.01, ****P* < 0.001.


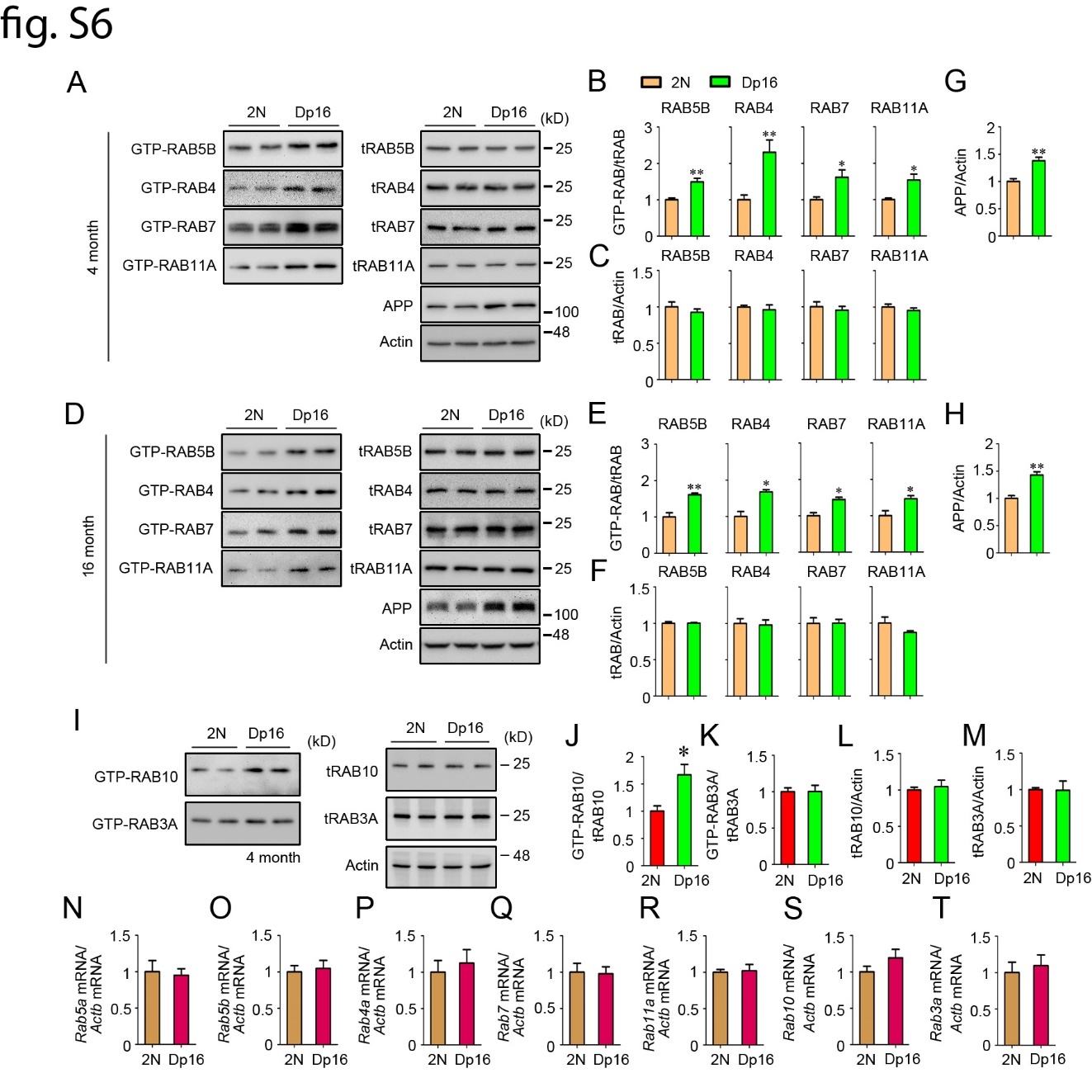


**Figure S6. Hyperactivation of endosomal Rabs in Dp16 brains.** (A, D) Western blot analysis of the levels of GTP-RAB5B, RAB4, RAB7, and RAB11A, and total Rabs in the brains of 4-month (A) and 16-month-old (D) Dp16 and 2N mice. APP was also probed with β-Actin as a loading control. (B, C and E, F) Quantitation and statistical analysis of the levels of GTP-Rabs (B, E) and total Rabs (C, F). (G, H) Quantitation and statistical analysis of APP levels. N = 5 in 4-month-old 2N and Dp16 for RAB5B, 4, and 7, n = 4 for RAB11A; n = 3 in 16-month-old 2N and Dp16 for RAB5B, 4, 7, and 11A. (I) The levels of GTP-RABs 10 and 3A and total RABs 10 and 3A, were measured in the brains of 4-month-old 2N and Dp16 mice. Quantitation and statistical analysis of the levels of GTP-RAB 10 and 3A (J, K) and total RAB 10 and 3A (L, M). N = 6 in 2N and Dp16 for GTP-RAB10 and GTP-RAB3A; n = 5 in 2N and Dp16 for tRAB10 and tRAB3A. (N-T) RT-PCR was used to analyze the mRNA levels of different Rabs in the brains of 4-month-old 2N and Dp16 mice. N = 7 in 2N, n = 5 in Dp16. Unpaired student *t*-test; **P* < 0.05, ***P* < 0.01.


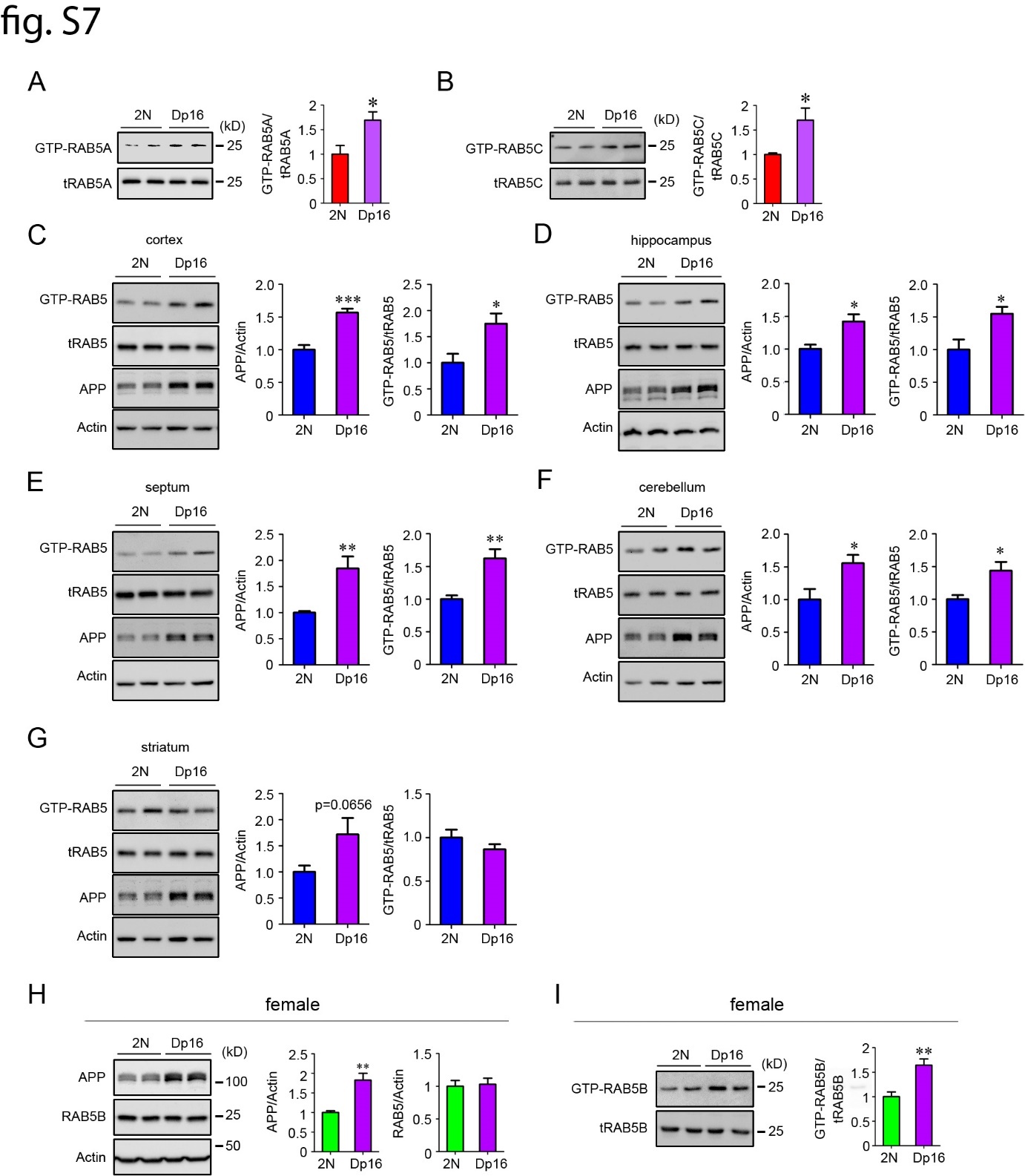


**Figure S7. Regional RAB5 hyperactivation in Dp16 brains.** (A, B) Isoform-specific antibodies were used to measure the levels of GTP-RABs 5A and 5C in the brains of 4-month-old Dp16 and 2N mice. N = 4 in 2N and Dp16 for RAB5A and RAB5C. (C-G) The activities of RAB5 were evaluated in the cortex (C), hippocampus (D), septum (E), cerebellum (F), and dorsal striatum (G) of 7-8-month-old Dp16 and 2N mice. N = 5 in 2N and Dp16 for cortex, hippocampus, cerebellum, and striatum; n = 5 in 2N, n = 4 in Dp16 for septum. (H) The levels of APP and RAB5B were measured in the cortex of 7-8-month-old female 2N and Dp16 mice with β-Actin as a loading control. (I) RAB5 activity was assessed in the cortex of 7-8-month-old female 2N and Dp16 mice. N = 5 in 2N and Dp16. Unpaired student *t*-test; **P* < 0.05, ***P* < 0.01, ****P* < 0.001.


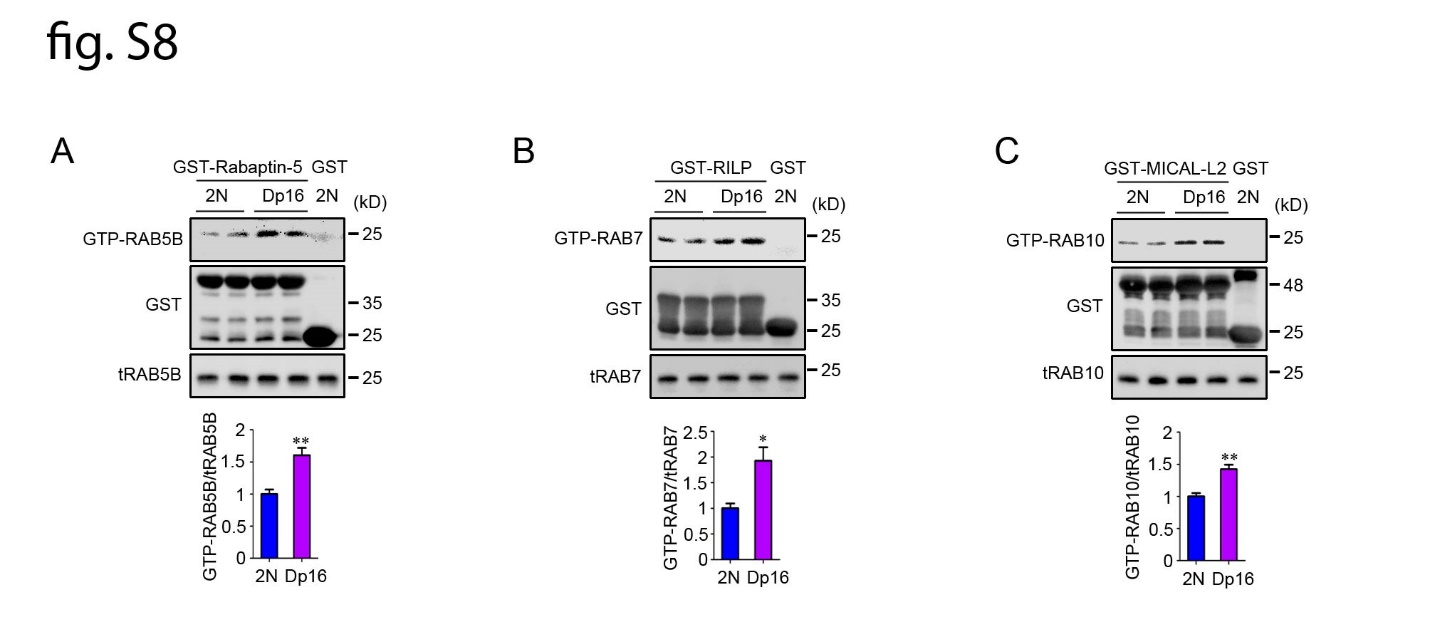


**Figure S8. Increased binding of endosomal Rab effectors with Rabs in Dp16 mice.** GST pull-down assays were used to evaluate the activities of RABs 5 (A), 7 (B), and 10 (C) with purified GST-Rabaptin-5, GST-RILP, and GST-MICAL-L2, respectively. GST was used as a control. N = 4 in 2N and Dp16. Unpaired student *t*-test; **P* < 0.05, ***P* < 0.01.


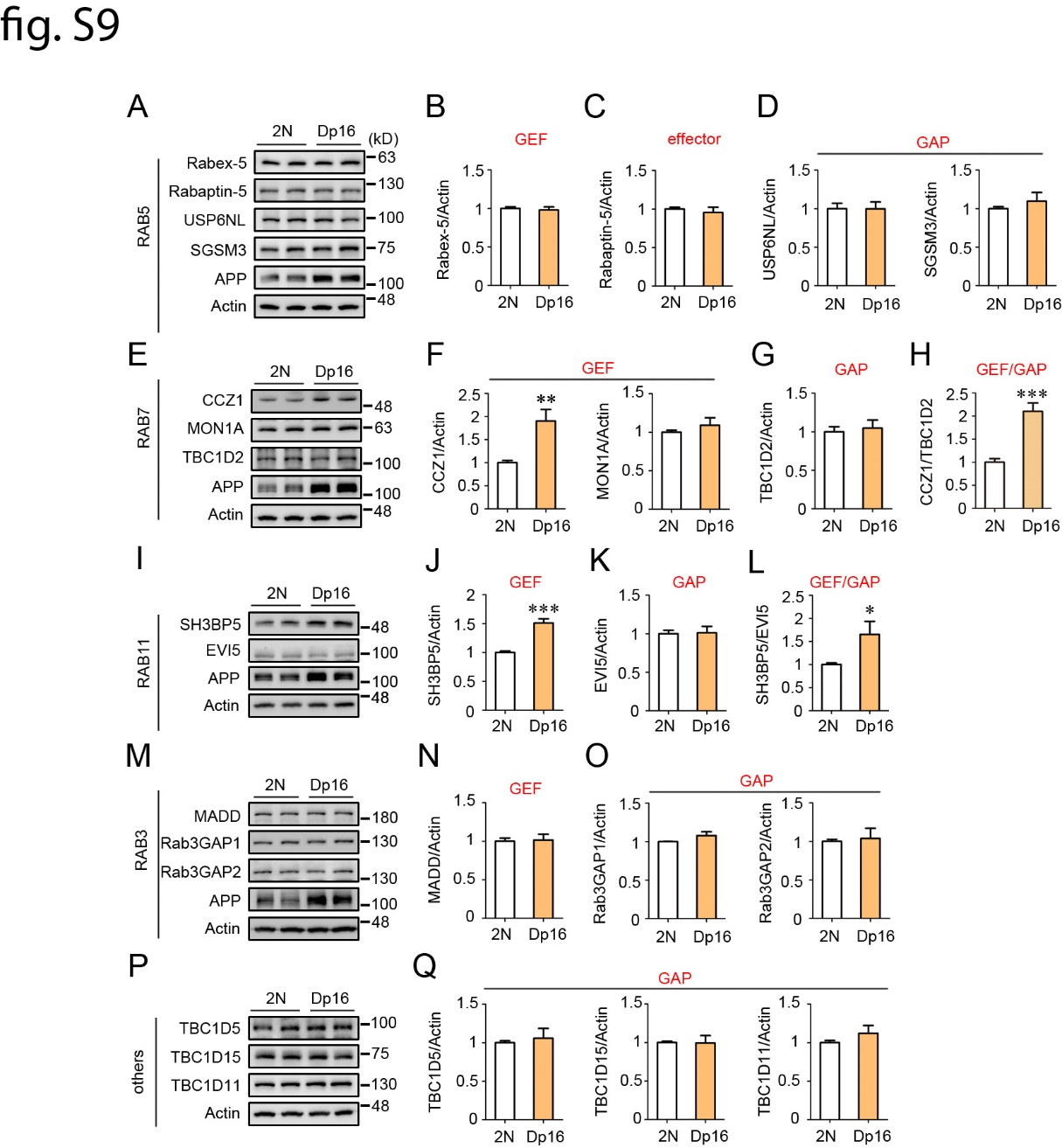


**Figure S9. Selective alterations of the GEFs of endosomal Rabs in the forebrains of Dp16 mice.** (A-D) The levels of GEFs/GAPs of RAB5 (A-D), RAB7 (E-H), RAB11 (I-L), and RAB3 (M-O) were measured in the brains of 4-month-old Dp16 and 2N mice. Quantitation and statistical analysis were displayed on the right panels. (P, Q) The levels of other GAPs were also analyzed in the 4-month-old 2N and Dp16 mice. N = 7 in 2N and Dp16 for Rabex-5; n = 6 in 2N and Dp16 for Rabaptin-5; n = 5 in 2N and Dp16 for USP6NL; n = 6 in 2N and Dp16 for SGSM3; n = 11 in 2N, n = 10 in Dp16 for CCZ1; n = 9 in 2N and Dp16 for MON1A; n = 9 in 2N, n = 8 in Dp16 for TBC1D2; n = 7 in 2N, n = 6 in Dp16 for CCZ1/TBC1D2; n = 13 in 2N and Dp16 for SH3BP5; n = 9 in 2N, n = 8 in Dp16 for EVI5; n = 7 in 2N, n = 6 in Dp16 for SH3BP5/EVI5; n = 5 in 2N and Dp16 for MADD and Rab3GAP1; n = 7 in 2N and Dp16 for Rab3GAP2; n = 5 in 2N and Dp16 for TBC1D5; n = 7 in 2N and Dp16 for TBC1D15 and TBC1D11. Unpaired student *t*-test for B, C; **P* < 0.05, ***P* < 0.01, ****P* < 0.001.


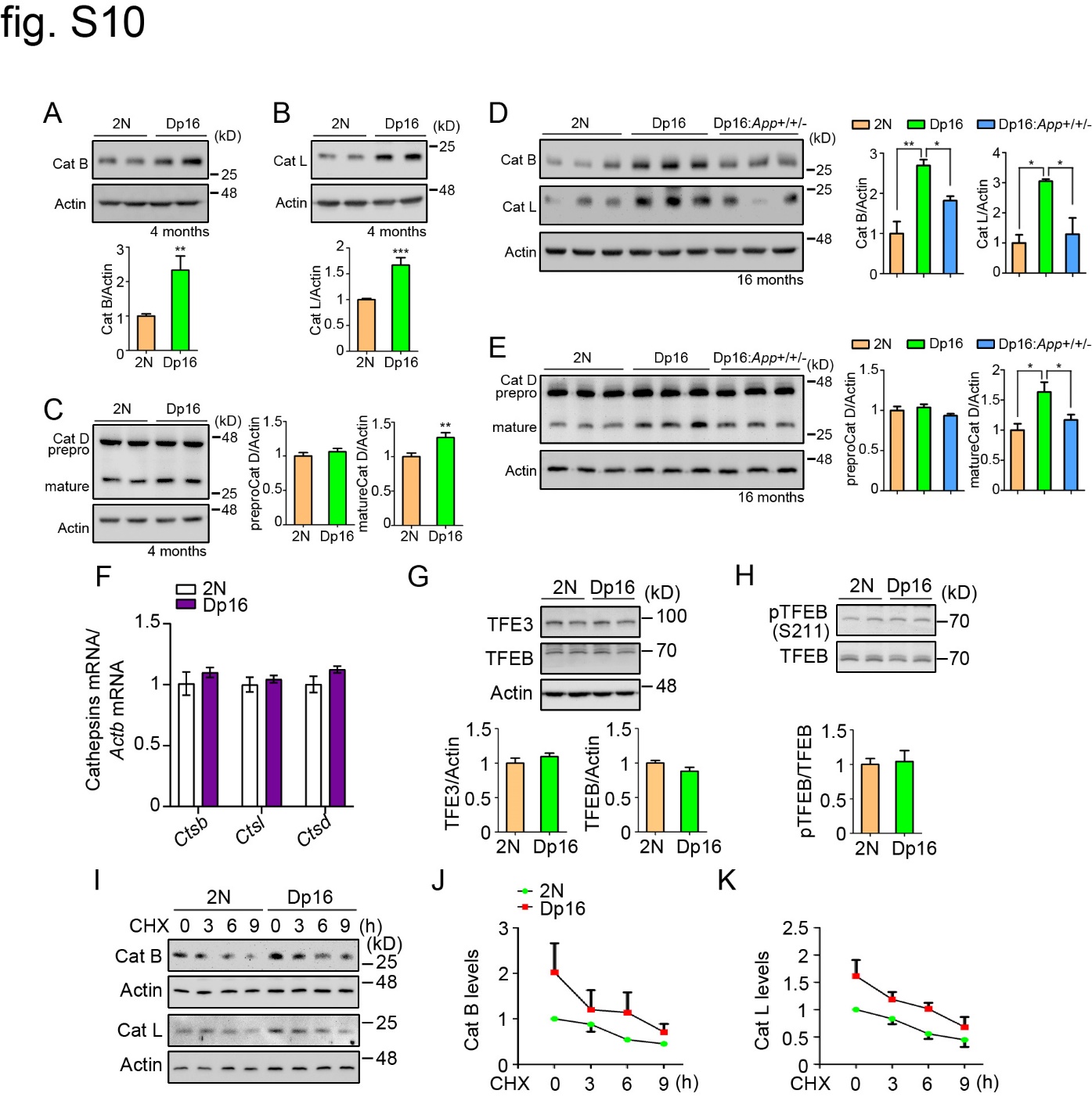


**Figure S10. APP-dependent changes in cathepsin levels in Dp16 brains.** (A-C) Western blot analysis of the levels of cathepsins B, L, and D in the brains of 4-month-old Dp16 and 2N mice. APP was also probed with β-actin as a loading control. N = 8 in 2N, n = 7 in Dp16 for Cat B; n = 10 in 2N and Dp16 for Cat L; n = 7 in 2N, n = 6 in Dp16 for Cat D. (D, E) The cathepsins B, L, and D levels were measured in brain homogenates from 16-month-old 2N, Dp16, and Dp16: *App*+/+/- mice with β-actin as a loading control. N = 3 in each genotype. (F) The relative mRNA levels of cathepsins in the cortex of 4-month-old 2N and Dp16 mice were assessed by qPCR. *Actb* mRNA was used as an internal control. N = 3 in 2N and Dp16 for each cathepsin. (G, H) Western blot analysis of the levels of transcription factors TFE3, TFEB, and pTFEB (S211) in the brains of 4-month-old Dp16 and 2N mice. N = 5 in 2N and Dp16. (I) Assessment of the decay rate of cathepsins B and L in 2N and Dp16 cortical neurons in the presence of 100 μg/ml CHX for the indicated durations. (J, K) Quantitation and statistical analysis of the levels of the decay rate of cathepsins in I. N = 4 in 2N and Dp16 for Cat B, n = 5 for Cat L. Unpaired student *t*-test for A to C, F, and G; one-way ANOVA followed by Newman-Keuls Multiple Comparison Test for D and E; **P* < 0.05, ***P* < 0.01, ****P* < 0.001.


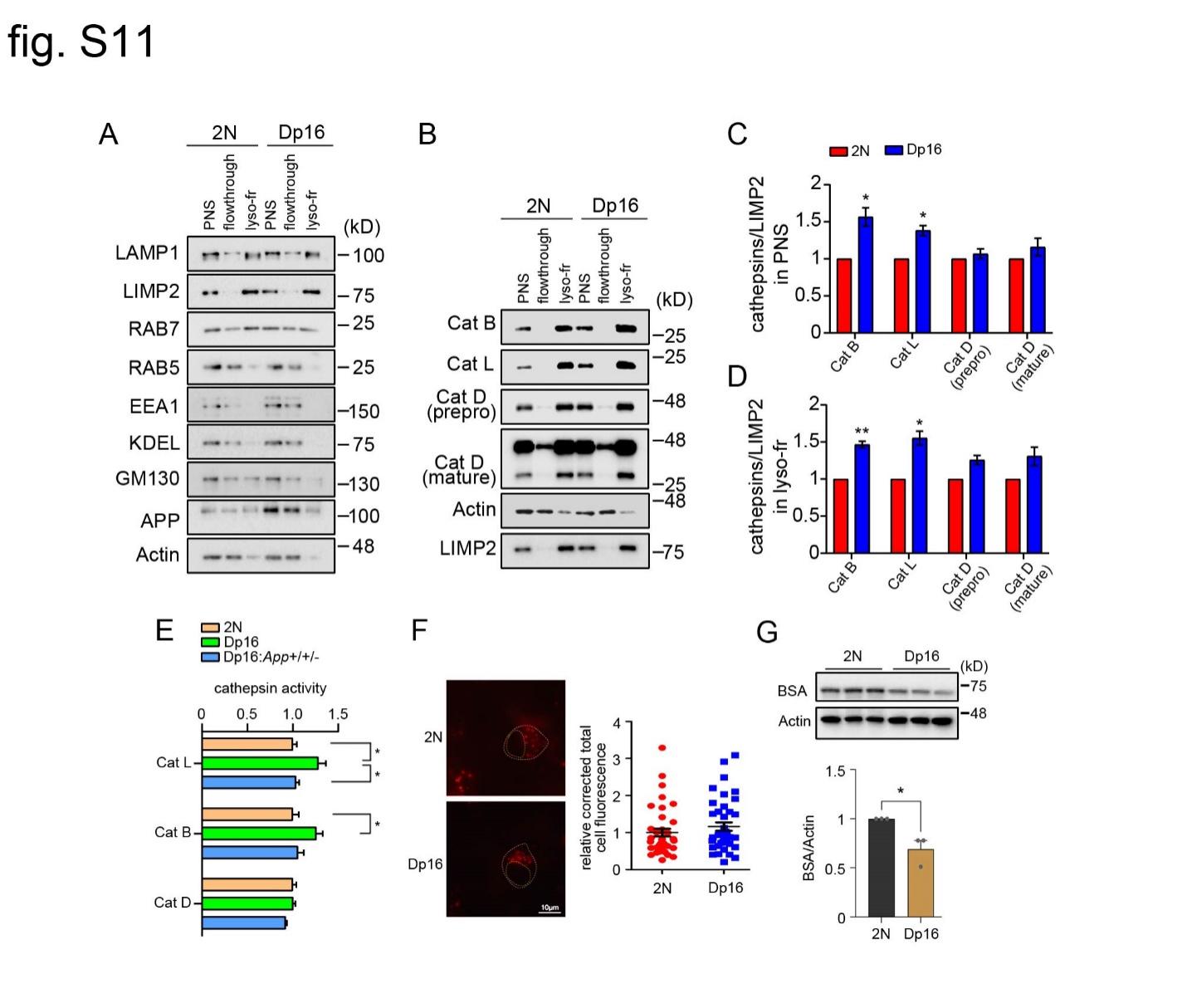


**Figure S11. Localization and activity of cathepsins and lysosomal degradative ability in Dp16.** (A) Evaluation of the efficiency of lysosome isolation from 2N and Dp16 neurons with dextran-coated magnetite beads. (B) Distribution of cathepsins in the magnetite beads-isolated lysosomes. (C, D) Quantitative analysis of cathepsins in postnuclear supernatants (PNS; C) and lysosome fraction (lyso-fr; D) normalized to the levels of lysosome marker LIMP2. N = 3 in 2N and Dp16 neurons. (E) The activities of cathepsins B, L, and D were measured in brain homogenates from 9-10-month-old 2N, Dp16, and Dp16: *App*+/+/- mice with individual kits. N = 5 in 2N, n = 5 in Dp16, n = 3 in Dp16: *App*+/+/- for Cat L; N = 6 in 2N, n = 6 in Dp16, n = 4 in Dp16: *App*+/+/- for Cat B; N = 4 in 2N, Dp16, and Dp16: *App*+/+/- for Cat D. (F) Live imaging of DQ-BSA in the primary cortical 2N and Dp16 neurons. Quantitative analysis of the corrected total cell fluorescence was shown on the right panel. Each dot represents the average value of puncta on focus in one cell. The experiment was repeated three times with more than ten fields captured each time. N = 40 neurons in 2N and Dp16. (G) Assessment of available DQ-BSA in the 2N and Dp16 neurons under the same setting as A. The experiment was repeated three times. Paired student *t*-test for B to D and G; one-way ANOVA followed by Newman-Keuls Multiple Comparison Test for E; unpaired student *t*-test for F; **P* < 0.05, ***P* < 0.01.


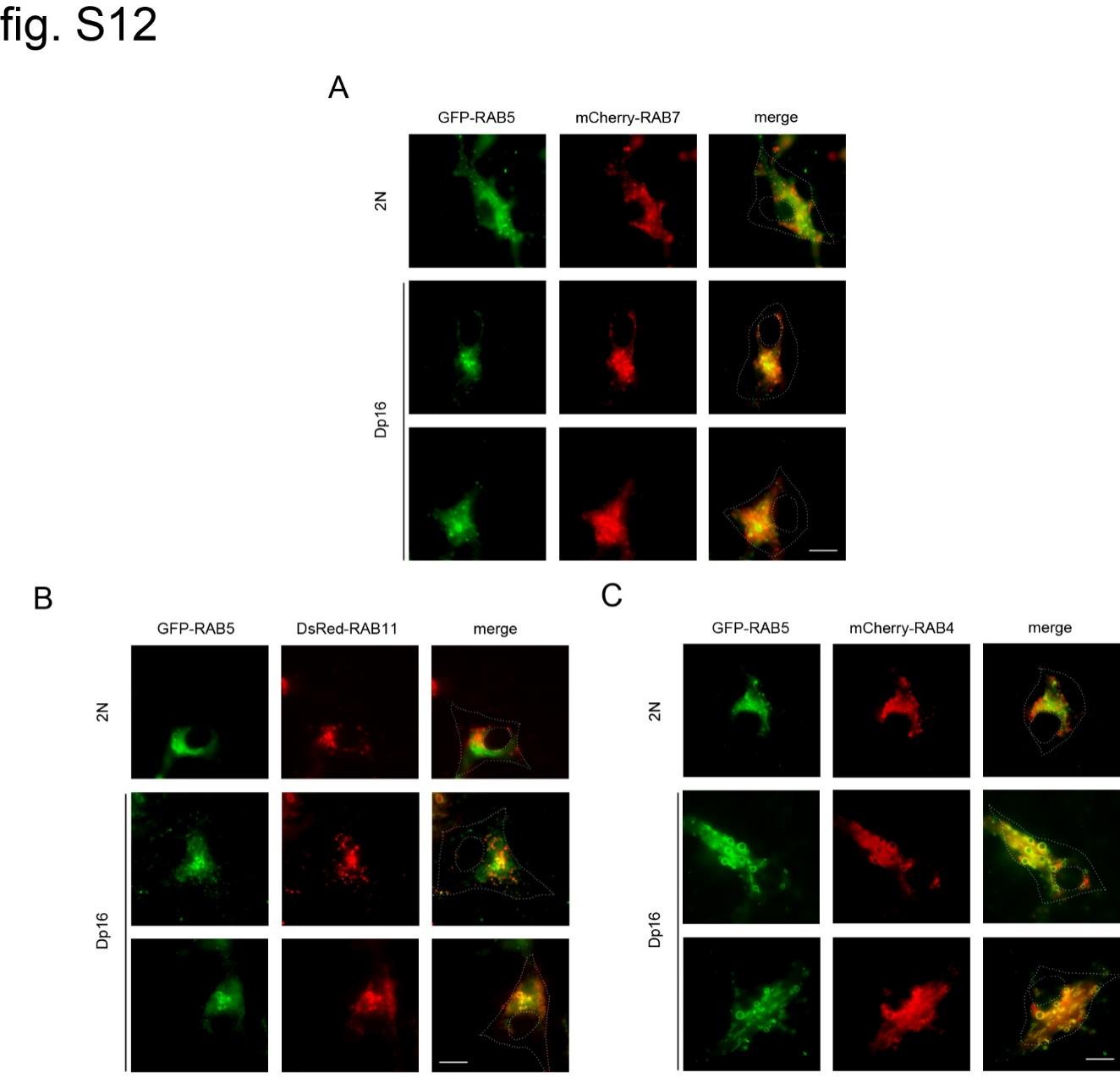


**Figure S12. Colocalization of RAB5 and other Rabs in 2N and Dp16 neurons.** (A) 2N and Dp16 neurons were infected with lentivirus expressing GFP-RAB5 and mCherry-RAB7 at DIV14 for 72 hours were live imaged. (B, C) 2N and Dp16 neurons were infected with lentivirus expressing GFP-RAB5 and DsRed-RAB11 or mCherry-RAB4 as A were live imaged. Dp16 neurons exhibited increased colocalization of transfected RAB5 with RABs 4, 7, and 11 compared to 2N controls. Scale bar = 10 µm.

Table S1. Summary of the mean ages, sex proportions, and *post-mortem* intervals between DS-AD, DS, AD, and their controls

|  | C/DS-AD (Mean ± SEM) | DS-AD (Mean ± SEM) | *P* value |
| --- | --- | --- | --- |
| Mean age (years) | 52.8 ± 1.3 | 53.7 ± 1.5 | 0.8991 |
| Sex proportion (F, %) | 50 | 50 |  |
| *post-mortem* intervals (hours) | 11.9 ± 1.5 | 10.6 ± 1.9 | 0.3261 |
|  | C/DS (Mean ± SEM) | DS (Mean ± SEM) | *P* value |
| Mean age (years) | 53.3 ± 1.1 | 40.3 ± 4.0 | 0.0139* |
| Sex proportion (F, %) | 50 | 50 |  |
| *post-mortem* intervals (hours) | 10.7 ± 1.8 | 15.4 ± 2.3 | 0.1253 |
|  | C/AD (Mean ± SEM) | AD (Mean ± SEM) | *P* value |
| Mean age (years) | 82.5 ± 4.4 | 82.3 ± 2.6 | 0.7422 |
| Sex proportion (F, %) | 45 | 50 |  |
| *post-mortem* intervals (hours) | 7.6 ± 3.2^#^ | 5.2 ± 2.1^#^ | 0.0372* |

^#^ analyzed with available recorded information; **P* < 0.05, Mann-Whitney tes­­­­­­­­t.

Table S2. Demographics, *post-mortem* interval, and clinical diagnosis for DS-AD, C/DS-AD, and PT-DS frontal cortex samples

| Patient | Gender | Age (Years) | *Post-mortem* interval (hours) | Clinical history consistent with DS-AD | Neuropathological findings consistent with DS-AD | Diagnosis |
| --- | --- | --- | --- | --- | --- | --- |
| C/DS-AD 1 * | Female | 49 | 7 | (-) | NP | Cognitively normal |
| C/DS-AD 2 * | Female | 50 | 7 | (-) | NP | Cognitively normal |
| C/DS-AD 3 * | Female | 46 | 18 | (-) | NP | Cognitively normal |
| C/DS-AD 4 * | Male | 48 | 6 | (-) | (-) | Cognitively normal |
| C/DS-AD 5 * | Male | 57 | 16 | (-) | NP | Cognitively normal |
| C/DS-AD 6 * | Male | 58 | 19 | (-) | NP | Cognitively normal |
| C/DS-AD 7 * | Female | 54 | 6 | (-) | NP | Cognitively normal |
| C/DS-AD 8 * | Female | 39 | 19 | (-) | NP | Cognitively normal |
| C/DS-AD 9 | Female | 59 | 5.9 | (-) | (-) | Cognitively normal |
| C/DS-AD 10 | Male | 59 | 5.9 | (-) | (+) Mild amyloid angiopathy | Cognitively normal |
| C/DS-AD 11 * | Male | 59 | 15.8 | (-) | (-) | Cognitively normal |
| C/DS-AD 12 * | Male | 58 | 9 | (-) | (-) | Cognitively normal |
| C/DS-AD 13 | Female | 48 | 8 | (-) | (-) | Cognitively normal |
| C/DS-AD 14 | Female | 51 | 21 | (-) | NP | Cognitively normal |
| C/DS-AD 15 | Female | 57 | 7 | (-) | (-) | Cognitively normal |
| C/DS-AD 16 | Male | 52 | 4 | (-) | (-) | Cognitively normal |
| C/DS-AD 17 | Male | 51 | 17 | (-) | (-) | Cognitively normal |
| C/DS-AD 18 | Male | 55 | 22 | (-) | (-) | Cognitively normal |
| Mean ± SEM | 9F/9M | 52.8 ± 1.3 | 11.9 ± 1.5 |  |  |  |
|  |  |  |  |  |  |  |
| DS-AD 1 | Female | 57 | 6 | (+) Dementia | (+) Brain atrophy; classic, extensive amyloid  deposition and neurofibrillary degeneration | Trisomy 21 with AD |
| DS-AD 2 * | Female | 51 | 4 | (+) Dementia | (+) Senile cerebral disease, Alzheimer’s type; congophilic angiopathy | Trisomy 21 with AD |
| DS-AD 3 * | Female | 46 | 7 | (+) Continued cognitive delay, declining verbal skill and functional status | (+) Diffuse and severe | Trisomy 21 with AD |
| DS-AD 4 | Male | 53 | 23 | NP | (+) AD neuropathological changes and cerebral amyloid angiopathy | Trisomy 21 with AD |
| DS-AD 5 * | Male | 55 | 12 | NP | (+) AD neuropathological changes and cerebral amyloid angiopathy | Trisomy 21 with AD |
| DS-AD 6 * | Male | 57 | 5 | NP | (+) Advanced AD (A3/B3/C3), cerebral amyloid angiopathy | Trisomy 21 with AD |
| DS-AD 7 * | Female | 55 | 25 | (+) | NP | Trisomy 21 with AD |
| DS-AD 8 | Male | 65 | 10 | (+) | (+) Generalized cerebral atrophy, diffuse and severe | Trisomy 21 with AD |
| DS-AD 9 | Male | 41 | 15 | (+) | (+) Advanced, Braak Stage VI, cerebral amyloid angiopathy | Trisomy 21 with AD |
| DS-AD 10 * | Male | 56 | 16 | (+) | (+) Advanced | Trisomy 21 with AD |
| DS-AD 11 * | Male | 64 | 20 | (+) MMSE 21/30 9 years before death | (+) Minimally atrophic brain with Alzheimer-type neuropathologic changes | Trisomy 21 with AD |
| DS-AD 12 | Female | 45 | 2.75 | (+) | (+) Braak Stage VI | Trisomy 21 with AD |
| DS-AD 13 | Female | 47 | 6.5 | (+) | (+) Braak Stage VI | Trisomy 21 with AD |
| DS-AD 14 | Female | 50 | 5 | (+) | (+) Braak Stage VI | Trisomy 21 with AD |
| DS-AD 15 | Female | 52 | 4.37 | (+) | (+) Braak Stage VI | Trisomy 21 with AD |
| DS-AD 16 | Female | 62 | 2.42 | (+) | (+) Braak Stage VI | Trisomy 21 with AD |
| DS-AD 17 * | Male | 53 | 24 | (+) | NP | Trisomy 21 with AD |
| DS-AD 18 | Male | 57 | 3 | (+) Alzheimer’s disease with early onset | NP | Trisomy 21 with AD |
| Mean ± SEM | 9F/9M | 53.7 ± 1.5 | 10.6 ± 1.9 |  |  | Trisomy 21 with AD |
|  |  |  |  |  |  |  |
| PT-DS | Male | 72 | 4.87 | (-) | (-) Only a single neuritic plaque and neurofibrillary degeneration consistent with normal aging but not AD | Partial trisomy 21 and cognitively normal |

Abbreviations: C/, control for; DS-AD, Alzheimer’s disease with Down syndrome; PT-DS, Down syndrome with partial trisomy 21; AD, Alzheimer’s disease; F, female; M, male; SEM, standard error of mean. *, samples utilized for RT-PCR analysis. A (+) sign in these columns indicates that the reported findings were consistent with a clinical history of cognitive decline or AD neuropathology, respectively; a (-) when the findings were inconsistent; and (NP: not provided) when no data were made available.

Table S3. Demographics, *post-mortem* interval, and clinical diagnosis for DS and C/DS frontal cortex samples

| Patient | Gender | Age (Years) | *Post-mortem* interval (hours) | Clinical history consistent with DS-AD | Neuropathological findings consistent with DS-AD | Diagnosis |
| --- | --- | --- | --- | --- | --- | --- |
| C/DS 1 | Female | 48 | 8 | (-) | NP | Cognitively normal |
| C/DS 2 | Female | 51 | 21 | (-) | NP | Cognitively normal |
| C/DS 3 | Female | 57 | 7 | (-) | (-) | Cognitively normal |
| C/DS 4 | Male | 52 | 4 | (-) | (-) | Cognitively normal |
| C/DS 5 | Male | 51 | 17 | (-) | (-) | Cognitively normal |
| C/DS 6 | Male | 55 | 22 | (-) | (-) | Cognitively normal |
| C/DS 7 | Female | 49 | 7 | (-) | NP | Cognitively normal |
| C/DS 8 | Female | 54 | 6 | (-) | NP | Cognitively normal |
| C/DS 9 | Female | 59 | 5.9 | (-) | (-) | Cognitively normal |
| C/DS 10 | Male | 48 | 6 | (-) | (-) | Cognitively normal |
| C/DS 11 | Male | 57 | 16 | (-) | NP | Cognitively normal |
| C/DS 12 | Male | 58 | 9 | (-) | (-) | Cognitively normal |
| Mean ± SEM | 6F/6M | 53.3 ± 1.1 | 10.7 ± 1.8 |  |  |  |
|  |  |  |  |  |  |  |
| DS 1 | Female | 50 | 23 | (-) | NP | Trisomy 21 without AD |
| DS 2 | Female | 39 | 12 | (-) | (-) | Trisomy 21 without AD |
| DS 3 | Male | 22 | 15 | (-) | NP | Trisomy 21 without AD |
| DS 4 | Male | 40 | 10 | (-) | Diffuse beta-amyloid deposition in the neocortex | Trisomy 21 without AD |
| DS 5 | Male | 25 | 22 | (-) | Rare neocortical diffuse plaques | Trisomy 21 without AD |
| DS 6 | Male | 25 | 24 | (-) | NP | Trisomy 21 without AD |
| DS 7 | Male | 57 | 22 | (-) | Frequent neuritic plaques, no significant neurofibrillary pathology | Trisomy 21 without AD |
| DS 8 | Male | 23 | 24 | (-) | NP | Trisomy 21 without AD |
| DS 9 | Female | 42 | 5 | (-) | Braak stage V | Trisomy 21 without AD |
| DS 10 | Female | 48 | 18.4 | (-) | Braak stage III | Trisomy 21 without AD |
| DS 11 | Female | 51 | 2.7 | (-) | Braak stage III | Trisomy 21 without AD |
| DS 12 | Female | 62 | 7 | (-) | Braak stage III | Trisomy 21 without AD |
| Mean ± SEM | 6F/6M | 40.3 ± 4.0 | 15.4 ± 2.3 |  |  |  |

Abbreviations: C/, control for; DS, Down syndrome without Alzheimer’s disease; AD, Alzheimer’s disease; F, female; M, male; SEM, standard error of mean. A (+) sign in these columns indicates that the reported findings were consistent with a clinical history of cognitive decline or AD neuropathology, respectively; a (-) when the findings were inconsistent; and (NP: not provided) when no data were made available.

Table S4. Demographics, PMI, and clinical diagnosis for AD and C/AD frontal cortex samples

| Patient | Gender | Age (Years) | *Post-mortem* interval (hours) | Diagnosis | Braak stage |
| --- | --- | --- | --- | --- | --- |
| C/AD-1 | Female | 75 | n/a | Cognitively normal | 1 |
| C/AD-2 | Male | 96 | 6 | Primary age-related tauopathy | 2 |
| C/AD-3 | Female | 96 | 6 | Cognitively normal | 2 |
| C/AD-4 | Female | 103 | 4 | Primary age-related tauopathy | 2 |
| C/AD-5 | Male | 84 | 36 | Cognitively normal | 2 |
| C/AD-6 | Male | 84 | 8 | Cognitively normal | 1 |
| C/AD-7 | Male | 79 | 3 | Cognitively normal | 2 |
| C/AD-8 | Male | 80 | 3.5 | Cognitively normal | 3 |
| C/AD-9 | Male | 93 | 3 | Cognitively normal | 1 |
| C/AD-10 | Female | 58 | 3.1 | Cognitively normal | 1 |
| C/AD-11 | Female | 59 | 3.1 | Cognitively normal | 1 |
| Mean ± SEM | 5F/6M | 82.5 ± 4.4 | 7.6 ± 3.2^#,^ * |  | 1.7 ± 0.2 |
|  | | | | | |
| AD-1 | Female | 100 | 5 | AD | 5 |
| AD-2 | Male | 75 | n/a | AD | 6 |
| AD-3 | Female | 74 | n/a | AD | 6 |
| AD-4 | Male | 79 | n/a | AD | 6 |
| AD-5 | Male | 73 | 5 | AD | 5 |
| AD-6 | Female | 85 | n/a | AD | 6 |
| AD-7 | Female | 91 | 24 | AD | 6 |
| AD-8 | Male | 64 | 2.3 | AD | 6 |
| AD-9 | Male | 74 | 2.6 | Probable AD | 6 |
| AD-10 | Male | 82 | 3 | Mixed vascular dementia | 5 |
| AD-11 | Male | 89 | 2.2 | Probable AD | 5 |
| AD-12 | Female | 84 | 2.5 | AD | 5 |
| AD-13 | Female | 89 | 2.7 | Probable AD | 6 |
| AD-14 | Female | 93 | 2.8 | Probable AD | 6 |
| Mean ± SEM | 7F/7M | 82.3 ± 2.6 | 5.2 ± 2.1^#^ |  | 5.6 ± 0.1 |

Abbreviations: C/, control for; AD, Alzheimer’s disease; F, female; M, male; SEM, standard error of mean. n/a, not available

Table S5. DNA primers and ASOs used in this study

| Primers for RT-PCR | | |
| --- | --- | --- |
| Primer name | Forward primer (5’-3’) | Reverse primer (5’-3’) |
| Mouse *Rab5a* | CAAGAACGGTATCATAGCTTAGCAC | CTTGCCTTTGAAGTTCTTTAACCC |
| Mouse *Rab5b* | GCAGGGAACAAAGCTGACCT | CTGGGGTTCGCTCTTTGG |
| Mouse *Rab4a* | CCATGTCCGAGACTTACGAT | CACCGACATTTATTATCTTTGAGCC |
| Mouse *Rab7* | ATGGTGGACGACAGACTTGT | CAACAAAAGGGAAGTTCTCGG |
| Mouse *Rab11a* | GCTGCTTTTCAGACAATTCTAACAG | ACTGCACCTTTGGCTTGTT |
| Mouse *Rab10* | GGCAAGACCTGCGTCCTTTT | GTGATGGTGTGAAATCGCTCC |
| Mouse *Rab3a* | ACCACAGCCTATTACCGAGG | GCATTGTCCCACGAGTAAGTTTT |
| Mouse *Ccz1* | AAGGACTTTTAGCCCATCGAAAC | CCGAACAACCATAACCATCCA |
| Mouse *Sh3bp5* | TATCAACCGACGGGAGACTGA | TGCCAGTTCGTCTAGTTTCAC |
| Mouse *Ctsb* | TCCTTGATCCTTCTTTCTTGCC | ACAGTGCCACACAGCTTCTTC |
| Mouse *Ctsl* | ATCAAACCTTTAGTGCAGAGTGG | CTGTATTCCCCGTTGTGTAGC |
| Mouse *Ctsd* | CCTGGCTTCGTCCTCCTTC | GGCGATGACTGCATGGAGT |
| Mouse *Actb* | GATCATTGCTCCTCCTGAGC | ACATCTGCTGGAAGGTGGAC |
| Human *RAB5A* | CAAGGCCGACCTAGCAAATAA | GATGTTTTAGCGGATGTCTCCAT |
| Human *RAB5B* | AGTCCTAGCATCGTTATTGCCC | ACTCCACCATACGTTTGTTGG |
| Human *RAB4A* | ATTGGAAATGCAGGAACTGG | CACGGACCTGAATCGTTCTT |
| Human *RAB7* | GTGTTGCTGAAGGTTATCATCCT | GCTCCTATTGTGGCTTTGTACTG |
| Human *RAB11A* | AGCGATGGCTGAAAGAACTGA | CCTGAGATGACGTAGATCACTCT |
| Human *RAB10* | CAAGGGAGCATGGTATTAGGTTT | CTAACGTGAGGAACGCCTTTT |
| Human *RAB3A* | GAGTCCTCGGATCAGAACTTCG | TGTCGTTGCGATAGATGGTCT |
| Human *CCZ1* | ACAAGGACATTTAGCCCATCAAA | GATTCCGAACAACCATGACCA |
| Human *SH3BP5* | TCCAGCTTTAGTTCAGGACCA | GCTGCATTCACTTCGAGGG |
| Human *CTSB* | GAGCTGGTCAACTATGTCAACA | GCTCATGTCCACGTTGTAGAAGT |
| Human *CTSL* | CTTTTGCCTGGGAATTGCCTC | CATCGCCTTCCACTTGGTC |
| Human *CTSD* | GAGCTGGTCAACTATGTCAACA | GCTCATGTCCACGTTGTAGAAGT |
| Human *ACTB* | GTCACACTTCATGATGGAGTTGAAGG | GACCTGACTGACTACCTCATGAAGAT |
| Primers for genotyping | | |
| *HPRT* | AGGATGTGATACGTGGAAGA | CCAGTTTCACTAATGACACA |
| *Il-2* | CTAGGCCACAGAATTGAAAGATCT | GTAGGTGGAAATTCTAGCATCATCC |
| *mApp* | AGAGCACCGGGAGCAGAGCG | AGCAGGAGCAGTGCCAAGC |
| *Neo* | ATGGATACTTTCTCGGCAGGAGC | GAGGCTATTCGGCTATGACTGGG |
| Primer for knockdown | | |
| Mouse *shAppl1* | CCGGCACACCTGACTTCGAAACTCTCGAG AGTTTCGAAGTCAGGTGTGTTTTTG | AATTCAAAAACACACCTGACTTCGAAACT CTCGAGAGTTTCGAAGTCAGGTGTG |
| *shLuc* | CCGGAGAATCGTCGTATGCAGTGAACTGCAG TTCACTGCATACGACGATTCTTTTTTG | AATTCAAAAAAGAATCGTCGTATGCAGTGAA CTCGAGTTCACTGCATACGACGATTCT |

Table S6. Demographics, *post-mortem* interval, and clinical diagnosis for DS-AD, C/DS-AD, DS, and C/DS frontal cortex samples used for GTP agarose pull-down assay

| Patient | Gender | Age (Years) | *Post-mortem* interval (hours) | Diagnosis |
| --- | --- | --- | --- | --- |
| C/DS-AD 1 | Female | 49 | 7 | Cognitively normal |
| C/DS-AD 2 | Female | 50 | 7 | Cognitively normal |
| C/DS-AD 3 | Female | 46 | 18 | Cognitively normal |
| C/DS-AD 4 | Male | 48 | 6 | Cognitively normal |
| C/DS-AD 5 | Male | 57 | 16 | Cognitively normal |
| C/DS-AD 6 | Male | 58 | 19 | Cognitively normal |
| C/DS-AD 7 | Female | 54 | 6 | Cognitively normal |
| C/DS-AD 8 | Female | 39 | 19 | Cognitively normal |
| C/DS-AD 9 | Female | 59 | 5.9 | Cognitively normal |
| C/DS-AD 10 | Male | 59 | 5.9 | Cognitively normal |
| C/DS-AD 11 | Male | 59 | 15.8 | Cognitively normal |
| C/DS-AD 12 | Male | 58 | 9 | Cognitively normal |
| DS-AD 1 | Female | 57 | 6 | Trisomy 21 with AD |
| DS-AD 2 | Female | 51 | 4 | Trisomy 21 with AD |
| DS-AD 3 | Female | 46 | 7 | Trisomy 21 with AD |
| DS-AD 4 | Male | 53 | 23 | Trisomy 21 with AD |
| DS-AD 5 | Male | 55 | 12 | Trisomy 21 with AD |
| DS-AD 6 | Male | 57 | 5 | Trisomy 21 with AD |
| DS-AD 7 | Male | 57 | 3 | Trisomy 21 with AD |
| DS-AD 8 | Female | 55 | 25 | Trisomy 21 with AD |
| DS-AD 9 | Male | 65 | 10 | Trisomy 21 with AD |
| DS-AD 10 | Male | 41 | 15 | Trisomy 21 with AD |
| DS-AD 11 | Male | 56 | 16 | Trisomy 21 with AD |
| DS-AD 12 | Male | 64 | 20 | Trisomy 21 with AD |
| DS-AD 13 | Male | 53 | 24 | Trisomy 21 with AD |
|  |  |  |  |  |
| C/DS 1 | Male | 57 | 6.7 | Cognitively normal |
| C/DS 2 | Male | 59 | 15.8 | Cognitively normal |
| C/DS 3 | Male | 52 | 4 | Cognitively normal |
| C/DS 4 | Male | 51 | 17 | Cognitively normal |
| C/DS 5 | Male | 55 | 22 | Cognitively normal |
| DS 1 | Male | 22 | 15 | Trisomy 21 without AD |
| DS 2 | Male | 40 | 10 | Trisomy 21 without AD |
| DS 3 | Male | 25 | 22 | Trisomy 21 without AD |
| DS 4 | Male | 25 | 24 | Trisomy 21 without AD |
| DS 5 | Male | 57 | 22 | Trisomy 21 without AD |

Table S7. Antibodies used in this study

| **Antibody** | **Vendor** | **Identifier** |
| --- | --- | --- |
| Rabbit polyclonal anti-RAB5B | Santa Cruz Biotechnology | Cat# sc-598; RRID: AB_2175453 |
| Mouse monoclonal anti-RAB4 | BD Biosciences | Cat# 610888; RRID: AB_398205 |
| Rabbit monoclonal anti-RAB7 | Cell Signaling Technology | Cat# 9367; RRID: AB_1904103 |
| Rabbit monoclonal anti-RAB11A | Abcam | Cat# ab128913; RRID: AB_11140633 |
| Rabbit monoclonal anti-APP/CTF | Abcam | Cat# ab32136; RRID: AB_2289606 |
| Mouse monoclonal anti-β-Actin | Proteintech | Cat# 60008-1-Ig; RRID: AB_2289225 |
| Mouse monoclonal anti-GST | Santa Cruz Biotechnology | Cat# sc-138; RRID: AB_627677 |
| Rabbit monoclonal anti-RAB10 | Cell Signaling Technology | Cat# 8127; RRID: AB_10828219 |
| Rabbit polyclonal anti-RAB3A | Cell Signaling Technology | Cat# 3930; RRID: AB_2177366 |
| Mouse monoclonal anti-RAB5A  (for IP and IP detection) | Synaptic Systems | Cat# 108111; RRID: AB_2619777 |
| Rabbit polyclonal anti-RAB5C | Abclonal | Cat# A7342; RRID: AB_2767879 |
| Rabbit monoclonal anti-DYRK1A | Cell Signaling Technologies | Cat# 8765; RRID: AB_2797660 |
| Rabbit monoclonal anti-Rabex-5 | Cell Signaling Technology | Cat# 7622; RRID: AB_10828937 |
| Mouse monoclonal anti-Rabaptin-5 | BD Biosciences | Cat# 610676; RRID: AB_398003 |
| Rabbit polyclonal anti-USP6NL | Bethyl Laboratories | Cat# A302-794A; RRID: AB_10631436 |
| Rabbit polyclonal anti-SGSM3 | Bethyl Laboratories | Cat# A305-661A; RRID: AB_2891570 |
| Mouse monoclonal anti-CCZ1 | Santa Cruz Biotechnology | Cat# sc-514290 |
| Rabbit polyclonal anti-MON1A | Abclonal | Cat# A17946; RRID: AB_2770385 |
| Rabbit polyclonal anti-TBC1D2A | LSBio | Cat# LS-C808380 |
| Rabbit polyclonal anti-SH3BP5 | GeneTex | Cat# GTX112002; RRID: AB_11166818 |
| Rabbit polyclonal anti-EVI5 (mouse) | MyBioSource | Cat# MBS9207132 |
| Rabbit polyclonal anti-EVI5 (human) | Bethyl Laboratories | Cat# A300-875A; RRID: AB_2101149 |
| Rabbit polyclonal anti-MADD | Bethyl Laboratories | Cat# A302-143A; RRID: AB_1720374 |
| Mouse monoclonal anti-Rab3GAP1 | Proteintech | Cat# 66688-1-Ig; RRID: AB_2882042 |
| Rabbit polyclonal anti-Rab3GAP2 | Proteintech | Cat# 24599-1-AP; RRID: AB_2879632 |
| Rabbit polyclonal anti-TBC1D5 | Proteintech | Cat#17078-1-AP; RRID: AB_2199389 |
| Rabbit polyclonal anti-TBC1D15 | Abclonal | Cat# A10593; RRID: AB_2758134 |
| Rabbit polyclonal anti-TBC1D11 | Abclonal | Cat# A15804; RRID: AB_2763226 |
| Rabbit polyclonal anti-APLP1 | Calbiochem | Cat# 171615; RRID: AB_10683250 |
| Rabbit polyclonal anti-APLP2 | Calbiochem | Cat# 171617; RRID: AB_565357 |
| Rabbit polyclonal anti-FLAG | Proteintech | Cat# 20543-1-AP; RRID: AB_11232216 |
| Mouse monoclonal TRKB | BD Biosciences | Cat# 610101; RRID: AB_397507 |
| Rabbit polyclonal anti-pTRKB (Tyr490) | Dr. Moses Chao (New York University) | N/A |
| Rabbit monoclonal anti-pAKT (Ser473) | Cell Signaling Technologies | Cat# 4060; RRID: AB_2315049 |
| Mouse monoclonal anti-AKT | Cell Signaling Technologies | Cat# 2920; RRID: AB_1147620 |
| Rabbit monoclonal anti-pERK1/2 (Thr202/Tyr204) | Cell Signaling Technologies | Cat# 4370; RRID: AB_2315112 |
| Rabbit monoclonal anti-ERK1/2 | Cell Signaling Technologies | Cat# 4695; RRID: AB_390779 |
| Rabbit monoclonal anti-pCREB (Ser133) | Cell Signaling Technologies | Cat# 9198; RRID: AB_2561044 |
| Rabbit monoclonal anti-CREB | Cell Signaling Technologies | Cat# 9197; RRID: AB_331277 |
| Mouse monoclonal anti-Syntaxin 1A | MilliporeSigma | Cat# S0664; RRID: AB_477483 |
| Rabbit polyclonal anti-SNAP25 | Proteintech | Cat# 14903-1-AP; RRID: AB_2192051 |
| Mouse monoclonal anti-PHF1 | Dr. Peter Davies (Albert Einstein University) | Cat# PHF1; RRID: AB_2315150 |
| Rabbit polyclonal anti-pTAU (Thr205) | Thermo Fisher Scientific | Cat# OPA1-03153; RRID: AB_326050 |
| Mouse monoclonal anti-TAU (TAU-5) | Thermo Fisher Scientific | Cat# AHB0042; RRID: AB_2536235 |
| Rabbit monoclonal anti-pGSK3β (Ser9) | Cell Signaling Technologies | Cat# 5558; RRID: AB_10013750 |
| Rabbit monoclonal anti-GSK3β | Cell Signaling Technologies | Cat# 12456; RRID: AB_2636978 |
| Rabbit anti-ASO backbone | Ionis Pharmaceuticals Inc | N/A |
| Rabbit monoclonal anti-RAB5 (for IP and IP detection) | Abcam | Cat# ab218624; RRID: AB_2892717 |
| Mouse monoclonal anti-RAB7 (for IP detection) | Cell Signaling Technologies | Cat# 95746; RRID: AB_2800252 |
| Mouse monoclonal anti-RAB11A (for IP detection) | Proteintech | Cat# 67902-1-Ig; RRID: AB_2918658 |
| Rabbit monoclonal anti-RAB5  (for IP detection) | Cell Signaling Technologies | Cat# 3547; RRID: AB_2300649 |
| Rabbit monoclonal anti-LAMP1 | Cell Signaling Technologies | Cat# 99437; RRID: AB_3065089 |
| Rabbit monoclonal anti-TFE3 | Cell Signaling Technologies | Cat# 81744 |
| Rabbit monoclonal anti-TFEB | Cell Signaling Technologies | Cat# 83010 |
| Rabbit monoclonal anti-pTFEB (Ser211) | Cell Signaling Technologies | Cat# 37681; RRID: AB_2799117 |
| Rabbit monoclonal anti-LIMP2 | Cell Signaling Technologies | Cat# 27960; RRID: AB_3083079 |
| Mouse monoclonal anti-EEA1 | MilliporeSigma | Cat# E7659; RRID: AB_10603495 |
| Mouse monoclonal anti-KDEL | Santa Cruz Biotechnology | Cat# sc-58774; RRID: AB_784161 |
| Mouse monoclonal anti-GM130 | Santa Cruz Biotechnology | Cat# sc-55591; RRID: AB_1124984 |
| Rabbit monoclonal anti-cathepsin B | Cell Signaling Technologies | Cat# 31718; RRID: AB_2687580 |
| Mouse monoclonal anti-cathepsin L (mouse) | Santa Cruz Biotechnology | Cat# sc-390367; RRID: AB_2827873 |
| Rabbit polyclonal anti-cathepsin L (human) | MyBioSource | Cat# MBS3013912 |
| Rabbit polyclonal anti-cathepsin D | Cell Signaling Technologies | Cat# 69854 |
| Mouse monoclonal anti-Bovine Serum Albumin (BSA) | Proteintech | Cat# 66201-1-Ig; RRID: AB_2881592 |
| Rabbit polyclonal anti-IBA1 | Proteintech | Cat# 10904-1-AP; RRID: AB_2224377 |
| Rabbit IgG control antibody | Proteintech | Cat# 30000-0-AP; RRID: AB_2819035 |
| Mouse IgG control antibody | Jackson ImmunoResearch Laboratories | Cat# 015-000-003; RRID: AB_2337188 |
| Goat anti-rabbit IgG-HRP | Jackson ImmunoResearch Laboratories | Cat# 111-035-144; RRID: AB_2307391 |
| Goat anti-mouse IgG-HRP | Jackson ImmunoResearch Laboratories | Cat# 115-035-003; RRID: AB_10015289 |
| Donkey anti-rabbit IgG Alexa Fluor™ 555 | Thermo Fisher Scientific | Cat# A-31572; RRID: AB_162543 |

Table S8. Summary of endolysosomal proteins in human *post-mortem* sample, Dp16 mouse, and in vitro Dp16 primary neurons

|  |  | DS vs. control | DS-AD vs.  control | PT-DS vs. control | Dp16 vs. 2N | Dp16: *App*++- vs. Dp16 | Dp16 neuron vs. 2N neuron | Dp16: RAB5^DN^ neuon vs. Dp16 neuron |
| --- | --- | --- | --- | --- | --- | --- | --- | --- |
| Rab proteins | RAB5B | No change | No change | No change | No change | No change | No change | ND |
|  | RAB7 | No change | No change | No change | No change | No change | No change | No change |
|  | RAB11A | No change | No change | No change | No change | No change | No change | No change |
|  | RAB4 | Increased | Increased | ND | No change | No change | ND | ND |
|  | RAB10 | Increased | Increased | No change | No change | No change | ND | ND |
|  | RAB3A | Increased | Increased | No change | No change | ND | N/A | ND |
| Specific Rab activity | RAB5B | Increased | Increased | No change | Increased | Reduced | Increased | ND |
|  | RAB7 | Increased | Increased | No change | Increased | Reduced | Increased | Reduced |
|  | RAB11A | Increased | Increased | No change | Increased | Reduced | Increased | Reduced |
|  | RAB4 | Increased | Increased | ND | Increased | Reduced | ND | ND |
|  | RAB10 | ND | No change | No change | Increased | Reduced | ND | ND |
|  | RAB3A | ND | No change | No change | No change | ND | ND | ND |
| GEF | Rabex-5  (RAB5) | No change | Reduced | ND | No change | ND | ND | ND |
|  | CCZ1  (RAB7) | Increased | Increased | No change | Increased | Reduced | Increased | Reduced |
|  | MON1A  (RAB7) | No change | No change | ND | No change | ND | ND | ND |
|  | SH3BP5  (RAB11) | Increased | Increased | No change | Increased | Reduced | Increased | Reduced |
|  | MADD  (RAB3) | No change | No change | ND | No change | ND | ND | ND |
| GAP | USP6NL  (RAB5) | Reduced | No change | ND | No change | ND | ND | ND |
|  | SGSM3  (RAB5) | No change | No change | ND | No change | ND | ND | ND |
|  | TBC1D2  (RAB7) | Reduced | Reduced | ND | No change | ND | ND | ND |
|  | EVI5  (RAB11) | Reduced | No change | ND | No change | ND | ND | ND |
|  | Rab3GAP1  (RAB3) | No change | No change | ND | No change | ND | ND | ND |
|  | Rab3GAP2  (RAB3) | No change | Reduced | ND | No change | ND | ND | ND |
|  | TBC1D11 | Reduced | Reduced | ND | No change | ND | ND | ND |
|  | TBC1D5 | Reduced | No change | ND | No change | ND | ND | ND |
| GEF/GAP | RAB5 | No change | No change | ND | N/A | ND | ND | ND |
|  | RAB7 | Increased | Increased | ND | Increased | ND | ND | ND |
|  | RAB11 | Increased | Increased | ND | Increased | ND | ND | ND |
|  | RAB3 | No change | No change | ND | No change | ND | ND | ND |
| Cathepsin protein | Cat B | Increased | Increased | No change | Increased | Reduced | Increased | Reduced |
|  | Cat L | Increased | Increased | No change | Increased | Reduced | Increased | Reduced |
|  | Cat D | No change | No change | ND | increased | Reduced | Increased | Reduced |
| Cathepsin activity | Cat B | ND | ND | ND | Increased | Reduced | Increased | Reduced |
|  | Cat L | ND | ND | ND | Increased | Reduced | Increased | Reduced |
|  | Cat D | ND | ND | ND | No change | No change | ND | ND |
| Lysosomal transcription factor | TFEB | ND | No change | ND | No change | ND | ND | ND |
|  | pTFEB (S211) | ND | No change | ND | No change | ND | ND | ND |
|  | TFE3 | ND | No change | ND | No change | ND | ND | ND |

ND, not done.
